# The contribution of latent factors of executive functioning to mind wandering: an experience sampling study

**DOI:** 10.1186/s41235-022-00383-9

**Published:** 2022-04-25

**Authors:** David Marcusson-Clavertz, Stefan D. Persson, Etzel Cardeña, Devin B. Terhune, Cassandra Gort, Christine Kuehner

**Affiliations:** 1 Department of Psychology, Linnaeus University, Hus L, Trummenvägen 11, 351 95 Växjö, Sweden; 2 CERCAP, Department of Psychology, Lund University, Lund, Sweden; 3 Department of Psychology, Goldsmiths, University of London, London, UK; 4 Research Group Longitudinal and Intervention Research, Department of Psychiatry and Psychotherapy, Central Institute of Mental Health, Medical Faculty Mannheim, Mannheim, Germany

**Keywords:** Mind wandering, Shifting, Updating, Inhibiting, Task-switching, Concentration, Guilt/fear-of-failure, Daydreaming, Working memory capacity, Ecological momentary assessments (EMA), Experience sampling method (ESM)

## Abstract

Accumulating evidence suggests that individuals with greater executive resources spend less time mind wandering. Independent strands of research further suggest that this association depends on concentration and a guilty-dysphoric daydreaming style. However, it remains unclear whether this association is specific to particular features of executive functioning or certain operationalizations of mind wandering, including task-unrelated thoughts (TUTs, comprising external distractions and mind wandering) and stimulus-independent and task-unrelated thoughts (SITUTs, comprising mind wandering only). This study sought to clarify these associations by using confirmatory factor analysis to compute latent scores for distinct executive functions based on nine cognitive tasks and relating them to experience sampling reports of mind wandering. We expected that individuals with greater executive control (specifically updating) would show a stronger reduction in SITUTs as momentary concentration and guilty-dysphoric style increase. A bifactor model of the cognitive battery indicated a general factor (common executive functioning) and ancillary factors (updating and shifting). A significant interaction between updating and concentration on mind wandering was observed with mind wandering defined as TUTs, but not as SITUTs (*N* = 187). A post hoc analysis clarified this discrepancy by showing that as concentration increases, both external distractions and mind wandering decrease more strongly among people with greater updating. Moreover, common executive functioning predicted a more negative slope of guilty-dysphoric style on SITUTs, whereas updating and shifting predicted more positive slopes. The opposite slopes of these executive functions on daily life mind wandering may reflect a stability-flexibility trade-off between goal maintenance and goal replacement abilities.

**Supplementary Information:**

The online version contains supplementary material available at 10.1186/s41235-022-00383-9.

## Significance statement

Mind wandering occurs during virtually every activity and people mind wander about one-third of their waking time. Although it often interferes with performance on current tasks, it may also serve positive functions, such as anticipating future scenarios. Improving our knowledge on mind wandering may help people minimize its costs and maximize its benefits. Two theoretical frameworks influenced this research. The control-failure hypothesis proposes that mind wandering occurs due to failures in attentional control abilities (i.e., executive functions), whereas the global availability hypothesis proposes that people with surplus attentional resources should have more resources available to mind wander more often. In addition, people differ greatly in their affective style of mind wandering, as some individuals typically experience more positive-constructive mind wandering, whereas others experience more guilty-dysphoric mind wandering, and these individuals may differ in how they control their mind wandering. In this paper, we discriminated between three executive functions, updating (i.e., temporarily storing and revising information in your memory), shifting (i.e., quickly switching between tasks), and common executive functioning (i.e., generally performing well on cognitive tasks, suggesting strong goal maintenance ability). We observed that these variables interacted differently with the guilty-dysphoric style of mind wandering and concentration on mind wandering. We argue that some of our findings support the global availability hypothesis, whereas others are more consistent with the control-failure hypothesis. Distinguishing between updating, shifting, and common executive functioning may help integrate these two accounts and increase our understanding of the role of attentional control in mind wandering.

## Introduction

When faced with distractions, humans can perform complex tasks by maintaining task-relevant information in working memory while filtering out task-irrelevant contents. For instance, at a party this ability enables us to follow a conversation while ignoring distracting conversations nearby. In situations that require us to focus our attention, we rely on a set of top-down processes referred to as *executive functioning*, which more generally override automatic behaviors (Diamond, 2013). Despite this ability to focus attention, humans spend on average at least about one-third of their waking states thinking about topics unrelated to their current activity (Kane et al., 2017), a phenomenon known as *mind wandering* (Smallwood & Schooler, 2015). Mind wandering is associated with poor performance during laboratory cognitive tasks (Randall et al., 2014) and daily life activities such as reading (Foulsham et al., 2013) and driving (Albert et al., 2018). Individual differences in executive functioning have been proposed to explain the highly variable amount of time people spend mind wandering in their daily lives. In particular, the control-failure hypothesis maintains that those with poorer executive functioning are less able to prevent mind wandering (McVay & Kane, 2010). Studies examining associations between latent factors have generally observed robust, negative correlations between executive functioning abilities and mind wandering measured in the laboratory—usually in the small-to-medium range (e.g., Kane et al., 2016; Unsworth & McMillan, 2017; Unsworth et al., 2021). However, the association between executive functioning and mind wandering measured in daily life using experience sampling methods appears to be very small and not robust (Kane et al., 2007, 2017; Marcusson-Clavertz et al., 2016).

One reason for the lack of robust associations between executive functioning and mind wandering in daily life may be that the latter has benefits in addition to the costs (Mooneyham & Schooler, 2013). For instance, mind wandering may facilitate planning the future, consolidating memories from the past, or engaging in rewarding fantasies (Klinger et al., 2018). Insofar as working memory is limited, mind wandering competes with task-related mentation for conscious accessibility and individuals with surplus cognitive resources may have more resources available to engage in mind wandering (i.e., the global availability hypothesis; Smallwood, 2010). Individuals may therefore show different inclinations to mind wander depending on the context (Robison & Unsworth, 2018; Robison et al., 2020; Smallwood & Andrews-Hanna, 2013). This interaction was supported by two experience sampling studies showing that people with superior executive functioning display a stronger decline in daily life mind wandering as concentration on the current task increases (Kane et al., 2007, 2017). That is, it appears that when the effort deployed to concentrate on the current task is high, people with greater executive functioning mind wander less, but when effort is low, they may mind wander *more* than those with poorer executive functioning (cf., Fig. 1 in Kane et al., 2017).

In addition to executive functioning and concentration predicting mind wandering, the contents of mind wandering mentation may be critical to understand how mind wandering is regulated. According to the content-regulation perspective, an adaptive cognitive system should be able to control the contents of self-generated thoughts, including mind wandering, to maximize the productivity of the experience (Smallwood & Andrews-Hanna, 2013). For example, future-oriented and goal-related mind wandering may support the pursuit of personal goals and problem-solving (Stawarczyk et al., 2011), whereas past-oriented negative mind wandering, such as ruminations on past stressors, may increase vulnerability for depression (Connolly & Alloy, 2018). Factor analytic work on individual differences in mind wandering have identified three factors referred to as daydreaming styles^1^ (Huba et al., 1982). Two of these styles primarily concern the affective content of the experience. Individuals who endorse a *positive-constructive* daydreaming style typically experience pleasant reactions during mind wandering, find them useful, and indicate that these episodes often contain vivid imagery, future temporal orientation, and creative problem-solving. In contrast, those who endorse a *guilty-dysphoric* daydreaming style typically experience guilty, hostile, and frightened reactions during mind wandering and these episodes often contain achievement-oriented and fear-of-failure fantasies. Individuals who endorse a *poor attentional control* style indicate that they easily get bored, have difficulty maintaining concentration, and quickly drift away from the subject. Whereas the positive-constructive style is associated with positive well-being, interest in introspection, and openness to experience, the guilty-dysphoric style is associated with negative well-being and neuroticism (Blouin-Hudon & Zelenski, 2016).

Consistent with the content-regulation perspective (Smallwood & Andrews-Hanna, 2013), one study indicated that affective daydreaming styles moderate the association between executive functioning and mind wandering in daily life (Marcusson-Clavertz et al., 2016). Specifically, among those with a greater guilty-dysphoric style, executive functioning was associated with less mind wandering, whereas this was reversed among those scoring lower in this style. In addition, the study observed an interaction between inhibition and the positive-constructive daydreaming style, indicating that mind wandering is more related to inhibition failures as this style decreases (see also Banks & Welhaf, 2021; Banks et al., 2016)*.* Taken together, these studies suggest that people with greater executive functions may more effectively control the amount of mind wandering depending on the effort to concentrate on the current task and the typical contents of mind wandering mentation. Common to these studies is a focus on working memory as an executive function and the use of complex span tasks to measure working memory (but see Kane et al., 2017). These tasks require multiple skills, including flexibly shifting between two subtasks, keeping to-be-remembered items updated in the mind, and inhibiting distractor items (cf., Miyake et al., 2000), prompting the question of which of these abilities predict the regulation of daily life mind wandering.

### Unity and diversity of executive functions

As executive functioning operates during a wide range of non-routine situations, such as when learning a new complex task, it requires several sub-abilities. These include monitoring of response schemas, inhibiting inappropriate schemas, and flexibly shifting to more appropriate ones (Norman & Shallice, 1986). One methodological approach to parsing these component processes has been to administer several cognitive tasks to tap these diverse functions and use factor analysis to examine their latent structure. An influential study administered a battery of nine tasks expected to differentially load on three executive functioning processes (Miyake et al., 2000). Examples of these tasks include the letter memory task to measure the ability to maintain and update item relations (*updating*), the stop-signal task to measure the ability to overcome prepotent responses (*inhibiting*), and the number-letter task to measure the ability to flexibly alternate between two subtasks (*shifting*). A correlated three factor model in which all latent factors were allowed to positively covary provided a good fit to the data and was selected as the optimal model (Miyake et al., 2000).

Although dozens of studies using this approach have tended to find support for the correlated three factor model, multiple studies have failed to identify the inhibiting factor (Friedman & Miyake, 2017). In reviewing this literature, Friedman and Miyake (2017) instead endorsed a bifactor model that includes a general factor (*common executive functioning*), with paths to all nine tasks, and two independent factors (*updating*-*specific* and *shifting*-*specific*), each with paths to three germane tasks. They argued that the correlated three factor model provides a similar fit as the bifactor model, but the latter simplifies interpretations when associating individual differences in these scores with other variables as common variance between the factors is partialled out. In the bifactor model, common executive functioning explains all the associations between the inhibiting tasks. Moreover, individual differences in common executive functioning and updating-specific ability are, as expected, strongly related to intelligence (Friedman et al., 2008), and the former is also strongly related to fewer attention-related problems during childhood as evaluated by teachers (Herd et al., 2014). By contrast, shifting-specific ability has shown a moderate *negative* association with intelligence (Friedman et al., 2008; Herd et al., 2014) and *positive* associations with attentional-related problems (Friedman et al., 2011; Herd et al., 2014), substance use (Gustavson et al., 2017), and procrastination (Gustavson et al., 2015), although these findings have resulted from mostly exploratory analyses and the negative intelligence correlation was not replicated in a recent study (Gustavson et al., 2022).

To explain the apparent opposite relations of common executive functioning and shifting-specific ability to intelligence and attention-related problems, Herd et al. (2014) adapted a neural network model focusing on the role of lateral prefrontal cortex and basal ganglia in executive functioning (Hazy et al., 2007). Herd and colleagues proposed that common executive functioning reflects goal maintenance, that is, the ability to bias attention towards goal-relevant representations so that weaker but more relevant stimulus–response mappings can overcome stronger, more habitual, ones when desirable. In this model, persistent neural firings in prefrontal cortex boost goal maintenance by increasing the signal-to-noise ratio of goal relevant information and stimulus–response mappings (see also Miller et al., 1996). Shifting-specific ability, on the other hand, may reflect goal-replacement skills. This ability is thought to be influenced by basal ganglia, which acts as a gating mechanism that determines whether new goal representations are allowed to enter prefrontal cortex by sending a “store” or “clear” signal to it. Shifting-specific ability is also thought to be influenced by activity in prefrontal cortex itself, but contrary to the common executive functioning factor, shifting is purported to be slowed down by persistent firings in prefrontal cortex, because they increase the “stickiness” of goal representations. These predictions were tested with a network simulation using the color-word Stroop task as an indicator of common executive functioning and the color-shape task as an indicator of shifting-specific ability. In support of their predictions, Herd et al. (2014) found that increasing the signal-to-noise ratio of activity in the layer corresponding to prefrontal cortex reduced the Stroop effect (suggesting enhanced common executive functioning) but increased the switch cost (suggesting diminished shifting). In addition, they found that manipulating the layer corresponding to basal ganglia, that is, the extent that this layer inhibits activity in prefrontal cortex influenced switch costs but not the Stroop effect. As the basal ganglia layer sent a greater “clearing” signal to the prefrontal cortex layer, switch costs decreased (suggesting enhanced shifting). These findings suggest that common executive functioning and shifting-specific abilities are dissociable phenomena that may sometimes show effects in the opposite directions of each other (see also Reineberg et al., 2018). The updating-specific ability was not tested in their model, but Friedman and Miyake (2017) proposed that it reflects the precision of the updating process, which may also be supported by basal ganglia insofar as it opens the gate to allow rapid updating of goal-relevant information in working memory and shuts the gate to prevent distractions from entering working memory (Frank et al., 2001).

### The relations of distinct executive functions to mind wandering in daily life

Despite multiple studies on the role of executive functioning in mind wandering (Kam & Handy, 2014; Randall et al., 2014), research on how specific latent factors relate to daily life mind wandering has been partly neglected. One exception is a study that assessed three latent factors labelled working memory capacity, attentional restraint (i.e., inhibiting a prepotent response), and attentional constraint (i.e., resolving interference from visual distractors) and related them to daily life mind wandering (Kane et al., 2017). Although none of these factors predicted overall mind wandering, working memory and attentional restraint abilities correlated with greater reductions in mind wandering as effort to concentrate on current activity increased. Thus, across two studies working memory capacity has moderated the relation between concentration and mind wandering (Kane et al., 2007, 2017). These studies have mainly used complex span tasks to measure working memory capacity, although the more recent study added two updating tasks. Complex span task performance correlates with updating, inhibiting, and shifting (Himi et al., 2019), but primarily loads on the updating factor (Miyake et al., 2000), suggesting that the relation between complex span performance and mind wandering may specifically generalize to the updating factor in the model proposed by Miyake et al. (2000). It is also possible that the finding concerning attentional restraint generalizes to the inhibiting/common executive functioning factor (Friedman & Miyake, 2004, 2017; Kane et al., 2016). Furthermore, the study that found working memory capacity to predict lower mind wandering as the guilty-dysphoric style increased used a symmetry span task to measure working memory (Marcusson-Clavertz et al., 2016), a task that correlates highly with updating tasks (Hartung et al., 2020). It is worth noting that this task loads strongly on both the working memory-specific and the common executive functioning factor in the model presented by Kane et al. (2016). As none of these studies evaluated shifting abilities or examined unique contributions of the factors proposed by Friedman and Miyake (2017), the specific relations of these cognitive functions to daily life mind wandering remain unclear.

### Operationalizing mind wandering

A further important consideration in the link between executive functioning and mind wandering is how the latter is operationalized. Researchers vary in how they operationalize mind wandering (Kane et al., 2021; Weinstein, 2018). Neuroimaging studies tend to operationalize it as *stimulus-independent thoughts* (SITs), that is, thoughts or images unrelated to current stimuli in the surroundings (e.g., Mason et al., 2007). This research has demonstrated that self-reported SITs are associated with activation of the default mode network (Mason et al., 2007), a set of brain regions activated during rest (Raichle, 2015). In contrast, studies using behavioral measures and explicit tasks often operationalize mind wandering as *task-unrelated thoughts* (TUTs). For example, this research has found that self-reported TUTs are associated with behavioral indices of reduced processing of task stimuli (Foulsham et al., 2013). Finally, some researchers have operationalized mind wandering as *stimulus-independent and task-unrelated thoughts* (SITUTs; Stawarczyk et al., 2011). This definition distinguishes mind wandering (e.g., remembering an old friend during a lecture) from task-unrelated but stimulus-dependent thoughts referred to as external distractions (e.g., being distracted by someone nearby talking on the phone), as well as stimulus-independent but task-related thoughts referred to as task-related interferences (e.g., evaluating one’s comprehension of the subject) and stimulus-dependent and task-related thoughts referred to as on-task focus (e.g., listening to the lecturer).

In support of the two-dimensional view of mind wandering, two studies used a factor analytic approach to argue that SITUTs and external distractions are dissociable phenomena (Unsworth & McMillan, 2014, 2017). SITUTs and task-related interferences also appear to be distinct (Frank et al., 2015). Another way to conceptualize mind wandering is to define it as a heterogeneous family of related phenomena, such as SITs, TUTs, and unguided thoughts, with graded membership from less to more prototypical mind wandering episodes (Seli et al., 2018). Seli et al. (2018) suggested that research on mind wandering variability should examine multiple dimensions of mind wandering to increase conceptual clarity. The experience sampling studies most relevant to the present one defined mind wandering as TUTs (Kane et al., 2007, 2017) and SITUTs (Marcusson-Clavertz et al., 2016).

A standard approach to measuring mind wandering is to use thought sampling probes in which participants are prompted to report whether they were mind wandering right before the probe. Several studies provide evidence for the validity of mind wandering probes. These studies have shown convergence of reports of TUTs with reduced neural processing of sensory stimuli (Braboszcz & Delorme, 2011), increased behavioral lapses (Foulsham et al., 2013), and worse task performance (Randall et al., 2014). Another study used principal components analysis to show that SIT and TUT reports load on one component characterized by spontaneous, fanciful, future-oriented, and past-oriented, but not present-oriented thoughts demonstrating both convergent and discriminant validity (Cardeña & Marcusson-Clavertz, 2016). Two studies reported the expected relations between daily life TUTs and contextual predictors (Kane et al., 2007, 2017). Studies that have measured mind wandering across multiple tasks or contexts have typically found experience sampling reports to be highly reliable (Kane et al., 2021).

### The present study

This study examined the unique relations of latent factors of executive functions to daily life mind wandering using a battery of nine laboratory tasks and intensive experience sampling across a week. An analysis plan was uploaded to the Open Science Framework platform prior to data collection (https://osf.io/hk4fc/).^2^ Data were collected at Lund University (Lund, Sweden) and the Central Institute of Mental Health (Mannheim, Germany) from November 2017 to October 2019. Some of the German data, relating procrastination and rumination to sleep and affect, has already been published (Gort et al., 2021), but the data reported in this paper are original.

We sought to conceptually replicate the findings that associations between working memory (updating) and mind wandering are moderated by concentration (Kane et al., 2007, 2017) and daydreaming style (Marcusson-Clavertz et al., 2016). These predictions are summarized in Table 1. In particular, we expected that as participants self-report deploying greater effort concentrating on a daily life current activity, those with superior updating would show a greater decline in mind wandering. However, because those measures may also tap inhibiting/common executive functioning, we also explored whether this effect was specific to updating or observed also for other executive functions.

**Table 1:** Summary of predictions of mind wandering (MW) and the previous research they were based on

Prediction	Previous research			
	Study	Finding	Operationalization	
			EF	MW
1. Executive functioning (specifically updating) predicts lower MW as concentration increases	Kane et al. (2007)	WMC predicts lower MW as concentration increases, *t*(122) = − 3.98***	Complex span tasks (*z*)	TUT
	Kane et al. (2017)^a^	WMC predicts lower MW as concentration increases, *N* = 274, *z* = − 3.39*** Attentional restraint predicts lower MW as concentration increases, *z* = − 3.77*** Attentional constraint predicts lower MW as concentration increases, *z* = − 2.59*^b^	Factor scores based on complex span and updating (working memory), restraint, and constraint tasks	TUT
2. Executive functioning (specifically updating) predicts lower MW as guilty-dysphoric style increases	Marcusson-Clavertz et al. (2016)	WMC predicts lower MW as guilty-dysphoric style increases, *t*(87) = − 2.90**	Symmetry span	SITUT
3. Executive functioning (specifically inhibiting/common executive functioning) predicts lower MW as positive-constructive style decreases	Marcusson-Clavertz et al. (2016)	High-congruency Stroop effect predicts higher MW as positive-constructive decreases, *t*(87) = − 1.99*	Stroop	SITUT

EF, Executive functioning; WMC, Working memory capacity; TUT, task-unrelated thought; SITUT, Stimulus-independent and task-unrelated thought

***p < .001, **p < .01, *p < .05

^a^This study evaluated the three EFs in separate models

^b^This *z*-score corresponds to a *p*-value of .01 but the study used a lower *α* threshold

We also expected two-way interactions between updating and guilty-dysphoric daydreaming style as well as inhibiting and positive-constructive daydreaming style on mind wandering. On the basis of previous research (Marcusson-Clavertz et al., 2016), we predicted that among those with a greater guilty-dysphoric style, updating would more negatively predict mind wandering whereas among those with a lower positive-constructive style, inhibiting (or common executive functioning) would more negatively predict mind wandering. We primarily operationalized mind wandering as SITUTs (Marcusson-Clavertz et al., 2016), but also operationalized it as TUTs to make our results more comparable with previous research on mind wandering and concentration (Kane et al., 2007, 2017). In accordance with Seli et al. (2018) we also examined mind wandering defined as SITs. In the analysis plan, we also proposed predictions about a set of six newly designed experience sampling items that might correspond to the inhibiting, updating, and shifting factors measured with cognitive tasks, but since updating and inhibiting correlated so highly in this dataset (0.84) and we endorsed a bifactor model with other latent factors, we found these predictions less relevant and report them in supplementary instead. We also had hypotheses about the relation between sleep and mind wandering, but these will be reported in a separate paper.

## Method

### Participants

Two hundred and two individuals participated in this study (18–42 years old, *M* = 24.95, SD = 5.11, 75 males). They were recruited at Lund University (Lund, Sweden; *n* = 139) and the Central Institute of Mental Health (Mannheim, Germany; *n* = 63). An a priori power analysis based on the previously observed two-way interaction between working memory and the guilty-dysphoric daydreaming style on mind wandering, indicated that with 70 observations per participants, 25% missing responses, and *α* = 0.05, a sample of 150 individuals would lead to over 80% power (*B* = − 0.27, SE = 0.09; Marcusson-Clavertz et al., 2016). This random intercepts analysis was performed in R (R Development Core Team, 2010) using the lme4 package (Bates et al., 2015) following a power simulation guide (Browne et al., 2009). Anticipating some attrition due to the experience sampling methodology and the large cognitive battery, we attempted to collect data from over 200 participants and terminated data collection on January 1, 2020, as planned. The study was advertised as evaluating attention, sleep, and daydreaming among individuals between 18 and 45 years old with advertisements placed mostly on noticeboards around the university campuses, and in online advertisements. The mean age was 25 years old for the Lund sample (67% females, 33% males) and 22 years old for the Mannheim sample (52% females, 48% males). The Lund sample comprised participants that studied full time (71%), worked full-time (9%), either studied or worked part-time only (12%), did not work or study (4%), or did not respond to the question (4%), whereas the Mannheim sample comprised individuals that studied full-time (40%), worked full-time (3%), worked or studied part-time only (55%), or did not work nor study (2%).

### Materials

#### Experience sampling methodology

Participants were given a digital wristband device (Pro diary, Camntech, Cambridge, UK) and frequently probed regarding their current attentional state. The experience sampling questionnaires were based on previous daily life research on mind wandering (Marcusson-Clavertz et al., 2016) and included 14 questions on thoughts, attention, and emotions and were administered in Swedish and German to the Lund and Mannheim samples, respectively. Table 2 shows an English translation of the Swedish questionnaire. We capitalized some words to make it easier for participants to quickly discriminate between items as the Pro-diary display is small and shows each question as a scrolling text. The German study appended two questions concerning rumination and procrastination at the end of the questionnaire (Gort et al., 2021). Mind wandering was primarily operationalized as any thought that was both task-unrelated and stimulus-independent (i.e., SITUTs; Stawarczyk et al., 2011), but we also examined mind wandering one-dimensionally as TUTs or SITs, respectively (see Table 2, questions 1 & 2). These two questions were also used to operationalize external distractions (thoughts related to the surroundings but unrelated to the current activity), task-related interferences (thoughts unrelated to the surroundings but related to the current activity), and on-task focus (thoughts related to both the surroundings and the activity).^3^

**Table 2:** Experience sampling questionnaire (translated from Swedish to English)

Item	Instruction/question	Response option
	Right before the beep…	
1	Were you thinking about the activity you were doing?	*Yes, activity* *No, something else*
2	Were you thinking about something in the immediate surroundings?	*Yes, surroundings* *No, something else*
3	Were you in control of/guiding your thoughts?	0 (*not at all*) to 1 (*fully*)
4	How aware were you of what you were thinking about?	0 (*not at all*) to 1 (*fully*)
5	How well can you remember what you were thinking about?	0 (*not at all*) to 1 (*very*)
6	How much were you trying to concentrate on the activity?	0 (*not at all*) to 1 (*a lot*)
7	Were you distracted by things in the immediate surroundings?	0 (*not at all*) to 1 (*a lot*)
8	Were you having difficulty maintaining concentration on what you were doing?	0 (*not at all*) to 1 (*a lot*)
9	Were you feeling…?	0 (*passive*) to 1 (*active*)
10	Were you feeling…?	0 (*sad*) to 1 (*happy*)
11	Were you feeling…?	0 (*anxious*) to 1 (*calm*)
12	Were you feeling…?	0 (*bored*) to 1 (*amused*)
13	In the last 10 min, were you having difficulty shifting focus between activities?	0 (*not at all*) to 1 (*a lot*)
14	In the last 10 min, were you having difficulty changing the way you thought about something?	0 (*not at all*) to 1 (*a lot*)

All 0–1 response scales were 100-point visual analog scales

We used question 6 as a measure of concentration, and the instructions clarified to participants that we were interested in the degree they were *trying* to concentrate, regardless of whether their thoughts actually were on task or not. A previous study used principal component analysis to show that this item loaded strongly on a component labelled focus/absorption (i.e., feeling active, interested, absorbed, and engaging in attention-demanding and interesting activities) and this component was distinct from the mind wandering component (Cardeña & Marcusson-Clavertz, 2016). In support of the validity of the measure, an unpublished result from a study administering the 2-back task indicated that blocks with greater concentration than person mean were accompanied by improved performance on the task, *B* = 0.13, SE = 0.01, *t*(10,582) = 16.90, *p* < 0.001 (a mixed model analysis; Marcusson-Clavertz et al., 2020).

#### The short imaginal processes inventory (SIPI)

The SIPI includes 45 items with three 15-item subscales (Huba et al., 1982). These subscales include the *positive-constructive* (e.g., “I daydream about what I would like to see in the future”), *guilty-dysphoric* (e.g., “My daydreams often contain depressing events which upset me”), and *poor attentional control* styles (e.g., “No matter how hard I try to concentrate, thoughts unrelated to my work always creep in”). Each item is answered on a Likert scale ranging from 1 (*definitely untrue or strongly uncharacteristic of me*) to 5 (*very true or strongly characteristic of me*). Item scores are summed to create subscales. The SIPI subscales have shown high test–retest reliability over 2–4 weeks (Marcusson-Clavertz & Kjell, 2019; Tanaka & Huba, 1986) and convergent and discriminant validity with trait questionnaires of spontaneous and deliberate mind wandering (Marcusson-Clavertz & Kjell, 2019). Unlike the poor attentional control style, the positive-constructive and guilty-dysphoric styles predicted the corresponding experience sampling scales (Marcusson-Clavertz et al., 2016) and did not significantly relate to the tendency to give socially desirable responses (Marcusson-Clavertz & Kjell, 2019).

#### Cognitive tasks

Nine tasks were chosen based on evidence from previous factor analytic models of executive functioning (Fisk & Sharp, 2004; Friedman et al., 2016; Miyake et al., 2000; Wolff et al., 2016). As planned prior to data collection, we used the linear integrated speed-accuracy score (LISAS) scoring computation (Vandierendonck, 2018) in four tasks with speed and accuracy outcomes (the flanker and the shifting tasks).^4^ This scoring approach takes both accuracy and speed of response into account measuring RTs adjusted for excessive errors. An individual LISAS score was computed for each task condition and participant as follows: (a) mean RT for correct responses is computed; (b) the SD of correct RTs is divided by the SD of the proportion of errors and then multiplied by the proportion of errors; (c) the sum of (a) and (b) is computed such that the adjusted RT is increased with greater errors (for more details, see Vandierendonck, 2018). Task switching scores were computed as switch LISAS minus repeat LISAS. All task outcomes were coded so that higher scores reflected better performance.

*Number-letter (shifting).* A sequence of number-letter pairs (e.g., G7) is shown in a counter-clockwise pattern across all four quadrants of the screen. Participants are required to alternate between identifying whether the letter is a consonant (press 1) or vowel (press 2) or the number is odd (press 1) or even (press 2) depending on whether the stimulus is shown in the upper (letter) or bottom (number) quadrants. Each number-letter pair is shown until a response is registered with a blank interstimulus interval of 150 ms. The task includes 127 experimental trials (64 repeat and 63 switch trials) divided in four blocks.

*Local–global (shifting).* A sequence of letters is shown with global and local features as each large letter is composed of smaller letters. Participants are required to alternate between indicating whether the large letter is an “S” (press 1) or “H” (press 2) or the small ones are “S” (press 1) or “H” (press 2) letters depending on whether the frame is blue (indicate the large letter) or green (indicate the small letters). The letter stimulus and the surrounding color frame is shown simultaneously up to 4000 ms or until a response is registered, followed by a 500 ms blank interstimulus interval. The task included 192 experimental trials, of which 64 were switch and 128 were repeat trials, divided in 5 blocks.

*Color-Shape (shifting).* A sequence of objects is shown including circles and triangles that are either green or blue. Participants are required to alternate between identifying whether the object is a circle (press 1) or triangle (press 2) or the color is blue (press 1) or green (press 2) depending on whether the cue word is “SHAPE” or “COLOR.” A cue word (“SHAPE” or “COLOR”) is shown 400 ms prior to each stimulus to indicate the task of the upcoming trial. The cue word is shown for 200 ms followed by a 200 ms blank screen. Subsequently the target stimulus is shown for 2000 ms or until a response is registered. The task included 180 experimental trials, of which 60 were switch and 120 were repeat trials, divided in 5 blocks.

*Flanker (inhibiting).* A sequence of arrows is presented, shown five at a time in one horizontal line, each arrow pointing left or right. In incongruent trials the central arrow points in the opposite direction to the others, whereas in congruent trials they all point in the same direction. Participants are required to indicate whether the central arrow points left (press 1) or right (press 2). Each trial begins with a fixation cross (450 ms) followed by a blank screen (450 ms), followed by target stimulus, which is shown up to 2500 ms or until a response is registered. The task includes 240 trials, of which 48 were incongruent and 192 were congruent, divided in 3 blocks. The outcome was a difference LISAS score (incongruent condition minus the congruent condition).

*Stop-signal (inhibiting).* A sequence of white squares and circles is shown. Participants are required to identify whether the object is a square (press 1) or circle (press 2) but withhold response on trials with an auditory stop signal (25% of the trials). The stimulus was shown for 1250 ms with the maximum RT also being 1250 ms. The auditory stop-signal begins with a 250 ms delay but is continuously adjusted to obtain a 50% stopping rate by decreasing 50 ms after unsuccessful stopping and increasing 50 ms after successful stopping. The task included 192 experimental trials divided in 3 blocks. If participants stop significantly more or less often than 50%, they are excluded. The outcome is the stop-signal RT, which is an estimate of the covert latency of the inhibition process, for which slower RTs reflect longer time required to successfully inhibit responses on stop-signals (Verbruggen et al., 2008).

*Go/No-go (inhibiting).* A sequence of letters is shown and participants are required to press button 1 for every letter they see (Go trials) except the letter X, for which they are required to withhold response (No-go trials). Each letter is shown for 500 ms with a 750 ms interstimulus interval (a fixation cross). The task included 256 trials, of which 50 were no-go trials and 206 were go trials, divided in 2 blocks. The planned outcome was arc sine transformed proportion of errors on No-go trials (commission errors), but because of issues of not obtaining a positive definite matrix and of fitting a bifactor model with a common executive functioning factor we used the broader SART index as the outcome to reflect broader failures to sustain attention. We found this index to be reliable in previous research (Marcusson-Clavertz et al., 2016), and there is theoretically driven research suggesting that these measures cover a wide range of inhibition failures ranging from micro-level fluctuations in RTs to complete lack of responding to the task. The SART index is the summation of standardized scores of commission errors on No-go trials and too fast responses (anticipations; RTs below 100 ms; rank scores to prevent outliers), RT coefficient of variability (SD/*M*), and the number of incorrect responses (omissions; rank scores) on Go trials (Cheyne et al., 2009; Marcusson-Clavertz et al., 2016).

*Keep-track (updating).* A sequence of words, each belonging to one of six categories, is presented on the monitor. In each trial, participants are required to keep track of the last word from certain target categories. The six categories include metals, fruits, animals, colors, relatives, and countries, and each category includes seven words. At the onset of every trial, the computer screen indicates the target categories for that trial (these are also shown during the remainder of the trial while words are presented). Each trial includes 15 words. Each word is shown for 1250 ms and is followed by a 250 ms interstimulus interval, which shows three asterisk signs. The subsequent recall screen prompts participants to type the last word of the target categories (no RT limit). There are four trials with 2, 3, and 4 target categories, respectively, so that raw scores range from 0 to 36. Task outcome score is the arc sine transformed proportion of correctly recalled target words (Miyake et al., 2000).

*Letter-memory (updating).* A sequence of letters is shown, and participants are required to update the last letters shown in correct position order. The letter pool includes all 26 letters of the English alphabet. Each trial presents 5, 7, 9, or 11 letters (in randomized order, but identical for all participants). Each letter is shown for 2000 ms without an interstimulus interval. In the first four trials participants are asked in advance to memorize the last three letters and in the last eight trials they are asked to memorize the last four letters. At the end of every trial, a prompt screen asks participants to type the correct letters in correct position. Raw scores range from 0 to 44. Task outcome score is the arc sine transformed proportion of correctly recalled target words (Miyake et al., 2000).

2*-back (updating)*. A sequence of letters is shown, and participants are required to recall whether the current letter matches the one shown two trials earlier. The letter pool includes B, F, H, K, M, Q, R, X, and Z. Each letter is shown for 500 ms followed by a 1000 ms blank screen. Participants press 1 if the current letter is a 2-back match and 2 if it is not. Each block begins with three practice trials. The task includes 148 experimental trials, 48 of which are 2-back matches and 100 that are not, divided into four blocks. The order was randomized within blocks for each participant. The number of 1-back or 3-back lures were not controlled for, but they were few in this dataset, approximately 2% of the non-target trials were 1-back lures and 4% were 3-back lures. Task outcome was the *d*’ sensitivity score, which is calculated by dividing the product of hit rate and correct rejection rate with the product of the false alarm rate and miss rate, and then transforming this score using the natural logarithm transformation.

#### Additional measures not included in this report

The pro-diary device also included data from an accelerometer and self-initiated morning and evening questionnaires about sleep. The Mannheim sample also included two experience sampling questions on procrastination (Gort et al., 2021). We also measured neuroticism, openness, dissociative tendencies, and physical activity with self-report questionnaires; these measures will be reported in a separate paper.

## Procedure

Participants were invited to take part in a one-week experience sampling study anchored by two laboratory sessions. In Session 1 participants provided informed written consent and completed four randomly chosen tasks from the nine-task battery. All tasks were presented in E-Prime software, version 3.0 (Psychology Software Tools, 2016), except for the Stop-signal task which was presented using an.exe file (Verbruggen et al., 2008). Participants responded using a keyboard for the letter-memory and keep-track tasks and a Cedrus response box (RB-740) for all other ones. In all tasks using the response box, participants were instructed to respond as accurately and fast as possible. The tasks were block-randomized for each participant so that each of three blocks included exactly one updating, one inhibiting, and one shifting task, and tasks of the same category could not follow each other. Task completion was interleaved with completion of psychometric measures including the SIPI. Participants were then given an instructional manual on the experience sampling device, the Pro-diary, and the experience sampling questionnaires. They were instructed to read the first four pages (covering the technical details and the TUT and SIT questions, including some examples) in the lab and the remainder at home. Each participant was asked to complete seven days of experience sampling with 10 programmed prompts per day in a 12.5-h window. At the end of session 1, participants selected the starting time of day to match their daily routine. Prompts were randomized within 10 blocks to reduce predictability (e.g., if a participant chose 9:00 as the starting time of day, the first prompt of the day would occur at a random time between 9:00 and 10:15). In addition to this probe-generated questionnaire, participants were asked to self-initiate a brief morning questionnaire when they awoke in the morning and a brief night questionnaire when they went to bed, but these questionnaires only addressed sleep-related questions and will be reported elsewhere. Participants completed two practice trials the evening before the onset of the seven-day experience-sampling period.

Participants were asked to return to the laboratory at their earliest convenience after the seven days of experience sampling. In session 2, participants completed the remaining five cognitive tasks interleaved with self-report measures. Participants were then debriefed and compensated. SDP ran the majority of the Lund sessions (DMC ran the remaining ones), whereas CG ran the Mannheim sessions.

## Analyses

All individual task and questionnaire outcomes that exceeded 3 SDs above or below the mean were replaced with the respective cut-off score to reduce the impact of outliers (Miyake et al., 2000). The number of participants with a corrected factor score was 1 for the common executive functioning factor, 1 for the updating factor, and 5 for the shifting factor, and 1 person also had a corrected score on the guilty-dysphoric style. That is, for each of these outcomes fewer than 3% of participants had corrected scores and their absolute *z* scores were below 4.12.

In order to examine the factor structure of the nine cognitive tasks, we performed a set of confirmatory factor analyses using SAS software, version 9.4 (SAS Institute, 2013). The analyses were performed using the Proc Calis technique. We used the full information maximum likelihood estimation method and set variance of each factor to 1 to simplify the interpretation of covariances. We compared a single-factor model, a three-factor model with correlated factors (Miyake et al., 2000), and a bifactor model with a common factor and two ancillary factors (Friedman & Miyake, 2017). We used the following indices and thresholds to estimate fit (Schreiber et al., 2006): to estimate the incremental fit of the hypothesized model compared to the baseline model, we used the Bentler comparative fit index (CFI) interpreting values greater than 0.95 as indicating good fit and greater than 0.90 as indicating acceptable fit. To estimate the absolute fit of the model, we used the Standardized Root Mean Square Residual (SRMR), interpreting values smaller than 0.05 and 0.08 as indicating good and acceptable fit, respectively. To estimate the parsimony of the model, we used the root mean square error of approximation (RMSEA), applying the same thresholds as for SRMR. We computed individual scores on each latent factor based on the endorsed model (using the Proc Score procedure).

In order to examine whether executive functioning and daydreaming styles predict daily life mind wandering, we performed multilevel modelling using HLM software, version 8.0 (Raudenbush et al., 2019). As planned, all intercepts and slopes were modelled as random, and the level-1 predictor (concentration) was centered within-person to examine cross-level interactions. We treated mind wandering reports at each prompt as level-1 observations nested within individuals (level-2). As mind wandering was coded as a binary variable, we used Bernoulli modelling with the Laplace estimation method (Raudenbush et al., 2019). Level-2 variables were standardized before computing interaction terms. We performed log likelihood tests based on the deviance statistics to compare hypothesized and baseline models. These multilevel modelling choices are the same as in a previous experience sampling study (Marcusson-Clavertz et al., 2016). The following analyses were planned prior to data collection.

Our first prediction was that executive functioning, particularly updating, predicts the slope of concentration on mind wandering. That is, those participants with greater updating were expected to show a steeper reduction in mind wandering as effort to concentrate on their current task increases. The baseline model included all latent executive functioning factors as level-2 predictors and concentration as the level-1 predictor of the outcome, whereas the confirmatory model added the updating factor as a predictor of the slope of concentration on mind wandering (i.e., a cross-level interaction). This prediction was tested by comparing the two models with a *χ*^2^ difference test (log likelihood). We also tested an exploratory model that examined whether adding the two remaining latent executive functioning factors to the slope of concentration on mind wandering would improve model fit. In addition to the planned analysis with mind wandering operationalized as SITUTs, we also examined these models with mind wandering operationalized as TUTs to conceptually replicate others’ research (Kane et al., 2007, 2017) and SITs to explore both parts of the two-dimensional operationalization.

The second prediction was that daydreaming styles and latent executive functioning factor(s) would interact in the prediction of mind wandering. First, we expected that among those higher in the guilty-dysphoric mind wandering style, updating should more negatively predict mind wandering. Second, we expected that among those lower in the positive-constructive style, inhibiting (or common executive functioning in case of a bifactor model) would be more negatively related to mind wandering. The baseline model included all relevant main effects (i.e., each of the latent executive functioning factors and the SIPI positive-constructive and guilty-dysphoric subscales). The confirmatory interaction model added two interaction terms, guilty-dysphoric × updating and positive-constructive × inhibiting (or common executive functioning, in the case of a bifactor model). Our prediction was tested by examining the *χ*^2^ difference test for these two models. We also tested an exploratory interaction model that added the remaining four interaction terms between the two SIPI mind wandering style subscales and the latent executive functioning factors. Again, our a priori operationalization was SITUTs but we also explored TUTs and SITs. In our analysis plan, we also proposed additional hypotheses that did not concern the relation between executive functioning and mind wandering (https://osf.io/hk4fc/) that are independent of the present analyses and will be evaluated in a separate paper. In addition to the planned analyses, we also performed a post hoc analysis that distinguished SITUTs from external distractions, task-related reappraisals, and on-task focus (Stawarczyk et al., 2011) by coding these four categories as one multinomial variable with on-task focus as the reference category (using robust standard errors). For inferential statistics, we set *α* to 0.05, two-tailed. Violin plots and line plots were created using GraphPad Prism version 9.0.0 (GraphPad Software, 2021).

## Results

### Data summary

Of the 202 participants who began the study, six withdrew during the experience sampling phase of the data collection. Three additional participants had missing scores on three or more cognitive tasks, resulting in a sample of 193 participants with cognitive data available for the confirmatory factor analysis. Due to technical errors, we could not retrieve the pro-diary data from six participants. Thus, the final analyses using both experience sampling and cognitive task outcomes were limited to 187 participants. The 15 participants with missing data did not significantly differ from the remainder of the sample on age, sex, or any of the SIPI dimensions (*p*s > 0.05). The final sample of 187 participants responded to 55.89 prompts (SD = 11.28) on average (out of 70), a response rate of 80%. Approximately 97% of the sample provided at least 30 reports each, with one participant providing only 7 reports (the remainder of the sample ranged from 20 to 70 reports). Omitting this participant did not have any noticeable impact on the results, so we included their data.

The final sample reported TUTs on 32% (SD = 17) of the prompts, which is similar to previous research (Kane et al., 2017). Similarly, participants reported SITs on 33% (SD = 17) of the prompts. Regarding the two-dimensional operationalization, participants reported SITUTs on 21% (SD = 13), external distractions on 11% (SD = 9), task-related interferences on 11% (SD = 13), and on-task focus on 57% (SD = 20) of the prompts, which is also similar to previous research (Marcusson-Clavertz et al., 2016; Stawarczyk et al., 2011). As for their co-occurrence, the TUTs included 67% SITUTs and 33% external distractions. In contrast, the task-related thoughts included 16% task-related interferences and 84% on-task focus. Person means of TUTs correlated strongly with SITs, *r*(185) = 0.61, *p* < 0.01, and SITUTs, *r*(185) = 0.86, *p* < 0.01. Mean concentration was 0.48 (SD = 0.12) on the 0 − 1 scale, suggesting that participants generally exerted medium effort to concentrate on their current activity. As for daydreaming styles, participants exhibited mean scores of 52.29 (SD = 8.04) on the positive-constructive style, 35.35 (SD = 9.25) on the guilty-dysphoric style, and 49.47 (SD = 9.25) on the poor attentional control style, which are similar to those reported in previous research (Huba et al., 1982; Marcusson-Clavertz & Kjell, 2019).

**Fig. 1:**
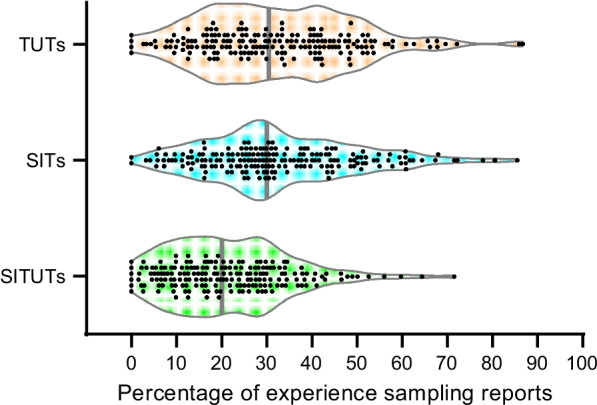
Violin plot showing the distributions of task-unrelated thoughts (TUTs), stimulus-independent thoughts (SITs) and stimulus-independent and task-unrelated thoughts (SITUTs) across individuals (*N* = 187). Vertical grey lines denote medians

The distributions of TUTs, SITs, and SITUTs for the final sample are shown in Fig. 1. Participant means of these variables showed small positive skewness values below 0.74. Skewness values were also small for SIPI daydreaming styles, with values between − 0.21 and 0.33. The outcomes of the nine cognitive tests showed skewness values between − 1.59 and 0.20. We considered these skewness values acceptable (see Additional file 1 for a descriptive summary of each of the nine tasks and each experience sampling question for the full sample).

### Confirmatory factor analyses and latent scoring of executive functioning

**Fig. 2:**
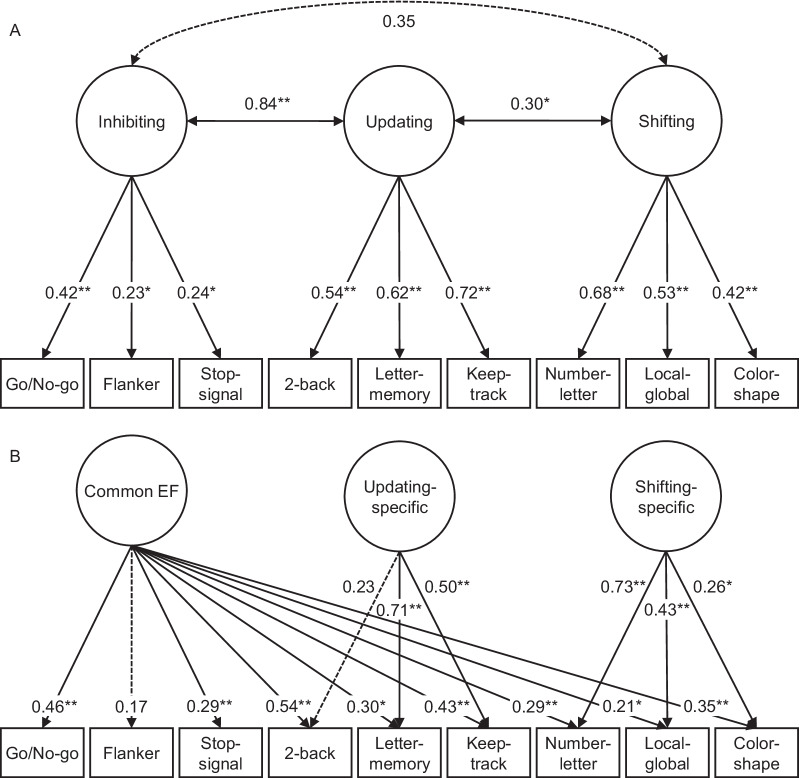
Illustration of path loadings for the correlated factors model (**A**) and the bifactor model (**B**) parametrizations (*N* = 193). EF = executive functioning. Solid lines represent significant loadings and dashed lines represent non-significant loadings. *p < .05, **p < .01

As expected, the one-factor model showed poor fit across all indices, *χ*^2^(27) = 72.67, *p* < 0.01, CFI = 0.73, SRMR = 0.08, and RMSEA = 0.09, indicating that the nine cognitive tasks did not measure a unitary factor. We next evaluated the correlated-factors model, according to which performance on the tasks is best described by three related but distinct latent factors (see Fig. 2A). The data deviated significantly from the model, *χ*^2^(24) = 37.71, *p* = 0.04, whereas fit indices indicated acceptable but not good fit, CFI = 0.92, SRMR = 0.06, RMSEA = 0.05. The correlation between the inhibiting and updating factors was extremely high (0.84) and all path loadings from the inhibiting factor were weak-to-moderate (see Fig. 2A), which is similar to previous research (Himi et al., 2019). Our results suggest that the updating and inhibiting factors were difficult to discriminate and that the inhibiting factor was not very well represented by the data.

Owing to the modest fit of the correlated-factors model, we next tested the bifactor model, which omits the inhibiting factor in favor of a common executive functioning factor and treats the factors as orthogonal. The data did not deviate significantly from this model *χ*^2^(22) = 25.27, *p* = 0.28, and showed good fit, CFI = 0.98, SRMR = 0.05, RMSEA = 0.03. However, the covariance matrix was not positive definite and there was an active boundary constraint for one of the nine tests (i.e., the error of the letter-memory task was set to 0). This may partly be due to the 2-back *d*-prime score having a surprisingly small non-significant path to the updating factor (standardized coefficient = 0.12, *p* = 0.29) resulting in the updating factor being driven primarily by letter memory task scores. Instead, the 2-back task showed a large significant loading path to the common executive functioning factor (0.62, *p* < 0.01), which was also highly driven by the go/no-go task (0.48, *p* < 0.01). We reasoned that although our planned outcome for the go/no-go task, commission errors, may be appropriate for measuring the inhibiting factor, as initially planned, a broader index of goal maintenance may better reflect the common executive functioning factor insofar as the latter reflects goal maintenance (Friedman & Miyake, 2017). For instance, omission errors can also be considered goal maintenance failures (Cheyne et al., 2009) even though they are clearly not indexing failures to inhibit a response. We therefore substituted commission errors in the go/no-go task for the SART index, which incorporates anticipations, RT coefficient of variability, omissions, and commission errors (Marcusson-Clavertz et al., 2016). The model exhibited good fit, *χ*^2^(21) = 25.89, *p* = 0.21, SRMR = 0.05, RMSEA = 0.03, CFI = 0.97, similarly to the aforementioned model but without the issue of an active constraint (see Fig. 2B for path loadings). Although the 2-back task still failed to load significantly on the updating factor, the standardized coefficient was now 0.23, *p* = 0.10, and the letter memory task no longer had an SE of 0. As this bifactor model was the best fitting of the tested models, consistent with recent research (Friedman & Miyake, 2017), and did not exhibit constraint issues, we computed scores for the three latent factors of this model for use in subsequent analyses. Factor scores were approximately normally distributed with acceptable skewness values in the final sample (common executive functioning: − 0.46; updating: 0.21; shifting: − 1.13). Table 3 shows the correlational data of the 193 individuals who contributed data to the factor analysis and the Cronbach’s α estimates of internal consistency. Reliability was generally above 0.70. The exceptions included the letter memory, keep-track, and the color-shape tasks, although error variance constituted less than half of the total variance for these tasks as well.

**Table 3:** Pearson correlations among extracted factor scores, cognitive tasks, and daydreaming styles (Cronbach’s *α* in diagonals)

Variables	1	2	3	4	5	6	7	8	9	10	11	12	13	14	15
1. Shifting-specific															
2. Updating-specific	− .16*														
3. Common EF	.22**	.32**													
4. Go/No-go^a^	.04	− .06	.62**	(*.80*)											
5. Flanker^b^	− .10	.12	.24**	.02	(*.73*)										
6. Stop-signal^c^	.03	.00	.40**	.11	.02	N/A									
7. 2-back^d^	− .06	.30**	.74**	.28**	.14	.19**	(*.87*)								
8. Letter memory^e^	− .10	.95**	.40**	.09	.15*	.09	.32**	(*.51*)							
9. Keep track^e^	.04	.66**	.58**	.13	.14*	.10	.34**	.48**	(*.62*)						
10. Number-letter^b^	.94**	− .11	.38**	.16*	.00	.11	.11	− .04	.12	(*.88*)					
11. Local–global^b^	.56**	.02	.27**	.13	− .15*	.06	.07	.06	.15*	.37**	(*.71*)				
12. Color-shape^b^	.32**	.06	.47**	.19**	.07	.12	.10	.16*	.21**	.29**	.18*	(*.59*)			
13. Positive-constructive	− .15*	− .08	− .01	.03	.07	.07	.02	− .08	− .06	− .15*	− .02	− .09	(*.80*)		
14. Guilty-dysphoric	.13	− .13	− .10	− .13	.10	.00	− .08	− .15*	− .08	.10	.05	− .07	.23**	(*.80*)	
15. Poor attentional control	− .03	− .02	− .13	− .09	− .03	.01	− .09	− .05	− .01	− .01	− .13	− .12	.09	.30**	(*.83*)

*N* = 193. Higher scores reflect better performance on all tasks

^a^SART index, ^b^LISAS score, ^c^Stop-signal reaction time, ^d^*d*’ score, ^e^Proportion of correctly recalled items arc sine transformed

### Predicting daily life experiences based on latent executive functioning factors

#### Evaluating the executive functioning × concentration hypothesis

For the 187 participants who contributed 10,257 observations for these analyses, we first performed a control analysis to check whether executive functioning predicted concentration, but the model including the three executive functioning factors did not outperform the null model, *χ*^2^(3) = 2.63, *p* = 0.45. Specifically, concentration was not significantly predicted by common executive functioning, *B* = − 0.01 (SE = 0.01), *p* = 0.09, updating-specific, *B* = 0.00 (SE = 0.01), *p* = 0.81, nor shifting-specific ability, *B* = − 0.00 (SE = 0.01), *p* = 0.83.

We tested the prediction that during moments when participants exerted greater effort to concentrate on their current task, those with greater executive functioning, particularly updating, would show a larger reduction in mind wandering. Table 4 shows the results for three operational definitions of mind wandering, namely SITUTs, TUTs, and SITs as outcomes. In brief, the results did not confirm this prediction with mind wandering operationalized as SITs or SITUTs, but there was support for it when mind wandering was operationalized as TUTs. Furthermore, regardless of how mind wandering was operationalized, none of the latent executive functioning factors significantly predicted the outcome, but concentration negatively predicted mind wandering across all models (see Table 4). That is, as participants reported exerting greater effort to concentrate on their current task, TUTs, SITs, and SITUTs strongly decreased, respectively.

**Table 4:** Results from multilevel models with executive functioning (EF) factors and momentary concentration as predictors of stimulus-independent and task-unrelated thoughts (SITUTs), task-unrelated thoughts (TUTs), and stimulus-independent thoughts (SITs)

	SITUTs				TUTs				SITs			
	*B* (SE)	*p*	OR	95% CI	*B* (SE)	*p*	OR	95% CI	*B* (SE)	*p*	OR	95% CI
*Null model (M*1*)*												
Intercept	− 1.46 (0.06)	< .01	0.23	[0.20, 0.26]	− 0.86 (0.06)	< .01	0.42	[0.37, 0.48]	− 0.82 (0.06)	< .01	0.44	[0.39, 0.50]
*Main effects model (M*2*)*												
Intercept	− **1.77 (0.08)**	** < .01**	**0.17**	**[0.15, 0.20]**	− 1.08 (0.08)	< .01	0.34	[0.29, 0.40]	− **0.96 (0.07)**	** < .01**	**0.38**	**[0.33, 0.44]**
Shifting-specific	− **0.06 (0.09)**	**.53**	**0.95**	**[0.79, 1.13]**	0.01 (0.09)	.93	1.01	[0.85, 1.20]	− **0.10 (0.09)**	**.25**	**0.90**	**[0.76, 1.08]**
Updating-specific	**0.09 (0.09)**	**.32**	**1.09**	**[0.92, 1.31]**	0.11 (0.08)	.18	1.11	[0.95, 1.30]	**0.05 (0.07)**	**.50**	**1.05**	**[0.91, 1.20]**
Common EF	− **0.00 (0.08)**	**.97**	**1.00**	**[0.85, 1.17]**	0.05 (0.10)	.57	1.06	[0.87, 1.28]	− **0.07 (0.08)**	**.41**	**0.94**	**[0.80, 1.10]**
Concentration	− **3.51 (0.20)**	** < .01**	**0.03**	**[0.02, 0.04]**	− 3.80 (0.19)	< .01	0.02	[0.02, 0.03]	− **2.16 (0.20)**	** < .01**	**0.12**	**[0.08, 0.17]**
*Confirmatory interaction model (M*3*)*												
Intercept	− 1.77 (0.08)	< .01	0.17	[0.15, 0.20]	− **1.08 (0.08)**	** < .01**	**0.34**	**[0.29, 0.40]**	− 0.96 (0.07)	< .01	0.38	[0.33, 0.44]
Shifting-specific	− 0.05 (0.09)	.54	0.95	[0.79, 1.13]	**0.01 (0.09)**	**.90**	**1.01**	**[0.85, 1.21]**	− 0.10 (0.09)	.25	0.90	[0.76, 1.08]
Updating-specific	0.07 (0.09)	.43	1.08	[0.90, 1.30]	**0.09 (0.08)**	**.26**	**1.09**	**[0.93, 1.28]**	− 0.00 (0.08)	.99	1.00	[0.86, 1.17]
Common EF	− 0.00 (0.08)	.97	1.00	[0.85, 1.17]	**0.05 (0.10)**	**.57**	**1.06**	**[0.87, 1.28]**	− 0.07 (0.08)	.42	0.94	[0.80, 1.10]
Concentration	− 3.50 (0.20)	< .01	0.03	[0.02, 0.05]	− **3.78 (0.19)**	** < .01**	**0.02**	**[0.02, 0.03]**	− 2.15 (0.20)	< .01	0.12	[0.08, 0.17]
Concentration × updating-specific	− 0.20 (0.20)	.32	0.82	[0.55, 1.22]	− **0.45 (0.21)**	**.03**	**0.64**	**[0.42, 0.96]**	− 0.33 (0.21)	.11	0.72	[0.48, 1.08]
*Exploratory interaction model (M*4*)*												
Intercept	− 1.76 (0.08)	< .01	0.17	[0.15, 0.20]	− 1.08 (0.08)	< .01	0.34	[0.29, 0.40]	− 0.96 (0.07)	< .01	0.38	[0.33, 0.44]
Shifting-specific	− 0.02 (0.09)	.85	0.98	[0.83, 1.17]	0.02 (0.09)	.83	1.02	[0.85, 1.22]	− 0.04 (0.09)	.67	0.96	[0.80, 1.16]
Updating-specific	0.08 (0.09)	.37	1.09	[0.90, 1.31]	0.09 (0.08)	.26	1.10	[0.93, 1.29]	0.02 (0.08)	.76	1.02	[0.88, 1.20]
Common EF	− 0.02 (0.08)	.80	0.98	[0.83, 1.16]	0.05 (0.10)	.59	1.05	[0.87, 1.28]	− 0.11 (0.09)	.19	0.89	[0.75, 1.06]
Concentration	− 3.49 (0.20)	< .01	0.03	[0.02, 0.04]	− 3.78 (0.19)	< .01	0.02	[0.02, 0.03]	− 2.14 (0.20)	< .01	0.12	[0.08, 0.17]
Concentration × shifting-specific	0.51 (0.23)	.03	1.67	[1.05, 2.64]	0.24 (0.22)	.28	1.27	[0.82, 1.98]	0.44 (0.22)	.05	1.55	[0.99, 2.41]
Concentration × updating-specific	− 0.05 (0.21)	.80	0.95	[0.63, 1.43]	− 0.39 (0.22)	.07	0.68	[0.44, 1.04]	− 0.16 (0.22)	.48	0.86	[0.56, 1.32]
Concentration × common EF	− 0.23 (0.22)	.31	0.80	[0.52, 1.23]	− 0.06 (0.22)	.78	0.94	[0.61, 1.45]	− 0.35 (0.21)	.11	0.71	[0.46, 1.08]

Model comparisons	Δ*χ*^2^	*p*			Δ*χ*^2^	*p*			Δ*χ*^2^	*p*		
*M*2 vs. *M*1 (*df* = 6)	**1194.34**	** < .01**			1728.71	< .01			**825.41**	** < .01**		
*M*3 vs. *M*2 (*df* = 1)	1.12	.29			**5.89**	**.02**			2.93	.08		
*M*4 vs. *M*3 (*df* = 2)	5.23	.07			1.14	.56			4.83	.09		
*M*4 vs. *M*2 (*df* = 3)	6.35	.09			7.03	.07			7.77	.05		

Endorsed models in bold. *N*_individuals_ = 187. *N*_moments_ = 10,257

With mind wandering operationalized as SITUTs, our a priori definition as employed in previous research (Marcusson-Clavertz et al., 2016), the main effects model provided significantly better fit than the null model (see Table 4). There was a significant intercept and a significant slope of concentration on SITUTs. The odds ratio of 0.17 for the intercept indicates that for average concentration and executive functioning, we expect 17 SITUTs for every 100 non-SITUTs. The odds ratio of 0.03 for concentration indicates that a 1-unit decrease (i.e., maximum reduction) in concentration is associated with 33 times greater odds of reporting a SITUT. Critically, the confirmatory interaction model, which included updating-specific ability as a predictor of the slope of concentration on SITUTs, did not significantly improve the fit over the main effects model. Thus, as shown in Table 4 we did not find support for our prediction that updating predicts the slope of concentration on SITUTs. Furthermore, the exploratory interaction model, which added shifting-specific and common executive functioning ability as predictors of the slope, did not significantly improve fit over the main effects model either. In sum, SITUTs decreased as concentration increased, but there was no support for the expectation that this relation would be moderated by executive functioning.

**Fig. 3:**
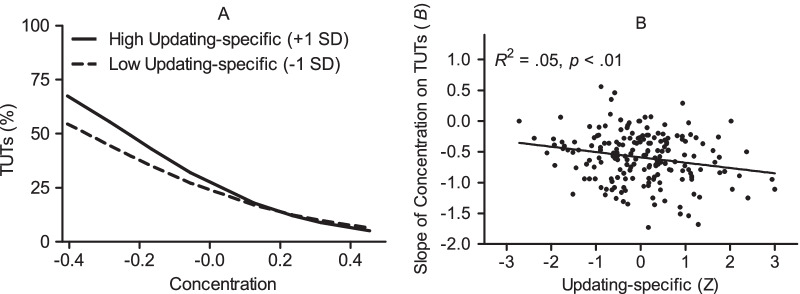
Task-unrelated thoughts (TUTs) as a function of the interaction between updating-specific ability and momentary effort to concentrate on one’s current task (*N* = 187): **A** The model based on the hierarchical linear modelling. **B** A scatterplot of updating-specific scores and slope coefficients of concentration on TUTs based on within-person ordinary logistic regression

With mind wandering operationalized as TUTs, as in previous research by other researchers (Kane et al., 2007, 2017), there was support for the prediction that updating relates to the slope of concentration on mind wandering. The results are shown in Table 4. First, the main effects model improved the fit compared to the null model. Adding updating-specific ability as a predictor of the slope of concentration on TUT improved the fit significantly compared to the main effects model. Updating-specific ability predicted a greater reduction in TUTs as concentration increased. Figure 3A illustrates this cross-level interaction between updating-specific ability and concentration on TUTs, which is similar to the pattern observed in a previous study (cf., Kane et al., 2017). The simple slopes analysis indicated that when participants were deploying effort to concentrate on their current task to a lesser degree than normal (− 1 SD), those with greater updating ability reported *more* task-unrelated thoughts, *B* = 0.22 (SE = 0.09), *p* = 0.01. This result is consistent with the global availability hypothesis (Smallwood, 2010). In contrast, when participants deployed greater effort to concentrate toward their current task (+ 1 SD), updating ability did not significantly predict mind wandering, *B* = − 0.04 (SE = 0.11), *p* = 0.73. To illustrate this finding with a scatterplot (see Fig. 3B), we performed ordinary logistic regression analyses with concentration as the predictor of TUTs for each participant separately and then correlated these within-person *B* coefficients with individual updating-specific scores. These slope coefficients of concentration on TUTs and updating-specific scores shared about 5% variance, *r*(185) = − 0.21, 95% CI [− 0.35, − 0.07], *p* < 0.01. Next, adding shifting-specific and common executive functioning abilities as predictors of the slope did not significantly improve the fit (see Table 4). To summarize these results, there was a small-to-medium sized interaction between updating-specific ability and momentary concentration on TUTs. As updating ability increases, the relation between concentration and TUTs becomes more strongly negative.

With mind wandering operationalized as SITs, updating-specific ability did not significantly predict the slope of concentration on SITs (see Table 4). The main effects model improved fit over the null model, but neither of the interaction models improved fit over the main effects model. To summarize, SITs were related to decreased concentration but not executive functioning, similar to the model of SITUTs.

As updating-specific ability significantly predicted the slope of concentration on TUTs, but not SITUTs, we conducted post hoc multinomial analyses to explore these effects further by discriminating SITUTs from external distractions, and task-related interferences as separate outcomes with on-task focus as the baseline. Updating-specific ability negatively predicted the slope of concentration on SITUTs, *B* = − 0.44 (0.19), *p* = 0.03, external distractions, *B* = − 0.60 (0.14), *p* < 0.01, and task-related interferences, *B* = − 0.43 (0.16), *p* < 0.01. This suggests that as effort increases, participants with greater updating-specific ability exhibit a stronger decrease in all other mentation categories compared to on-task focus. SITUTs, external distractions, and task-related interferences did not differ significantly from each other (*p*s > 0.25). This indicates that updating specifically predicted the slope of concentration on *on-task focus* (i.e., task-related *and* stimulus-dependent thoughts), whereas the other categories did not differ from each other. In other words, the moderating role of updating on the concentration–mind wandering association is not SITUTs-specific.

#### Evaluating the executive attention × daydreaming style hypothesis

Our second set of predictions concerned whether daydreaming styles would moderate the relation between latent executive functions and mind wandering. Specifically, we expected a two-way interaction between working memory updating and guilty-dysphoric daydreaming style on mind wandering. We further predicted a two-way interaction between inhibiting (or common executive functioning) and positive-constructive daydreaming style on mind wandering (Marcusson-Clavertz et al., 2016). As in our previous study, we primarily operationalized mind wandering as SITUTs (Marcusson-Clavertz et al., 2016), but we also explore the results for TUTs and SITs. In brief, the results did not support these two hypothesized interactions, but the planned exploratory analysis yielded significant interactions between the guilty-dysphoric style and all three executive cognitive factors on SITUTs.

**Fig. 4:**
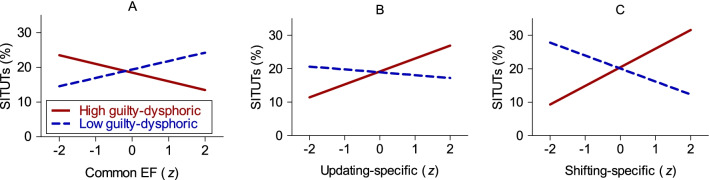
The percentage of stimulus-independent and task-unrelated thoughts (SITUTs) as a function of guilty-dysphoric style (± 1 SD) and **A** common executive functioning (EF), **B** updating-specific, and **C** shifting-specific abilities (*N* = 187)

The main effects model of SITUTs significantly improved the fit beyond the null model (see Table 5). The only independently significant predictor in this model was positive-constructive daydreaming style, which positively predicted daily life mind wandering. The odds ratio of 1.21 indicates that individuals with 1 SD greater positive-constructive style have 21% greater odds of reporting a SITUT. However, adding the two hypothesized interaction-terms did not significantly improve model fit, thus failing to support our prediction. In contrast, the exploratory interaction model, which added the remaining four interaction terms, improved the fit significantly compared to both the null model and the main effects model. As shown in Table 5, there was a negative two-way interaction between common executive functioning ability and the guilty-dysphoric style on SITUTs, similarly to previous research using a complex span task (Marcusson-Clavertz et al., 2016). As the guilty-dysphoric style increased by a standard deviation, the slope of common executive functioning on mind wandering became more negative by 0.16 log odds units (see Fig. 4). Simple slopes analyses did not yield any independently significant relation at ± 1SD with *B* = − 0.17 (SE = 0.11), *p* = 0.11 among those with a high guilty-dysphoric style (+ 1 SD) and *B* = 0.16 (SE = 0.10), *p* = 0.10 among those with a low guilty-dysphoric style (− 1 SD).

**Table 5:** Results from multilevel models with executive functioning (EF) factors and daydreaming stylesas predictors of mind wandering

	SITUTs				TUTs				SITs			
	*B* (SE)	*p*	OR	95% CI	*B* (SE)	*P*	OR	95% CI	*B* (SE)	*p*	OR	95% CI
*Null model (M*1*)*												
Intercept	− 1.46 (0.06)	< .01	0.23	[0.20, 0.26]	− 0.86 (0.06)	< .01	0.42	[0.37, 0.48]	− **0.82 (0.06)**	** < .01**	**0.44**	**[0.39, 0.50]**
*Main effects model (M*2*)*												
Intercept	− 1.47 (0.06)	< .01	0.23	[0.20, 0.26]	− 0.86 (0.06)	< .01	0.42	[0.37, 0.48]	− 0.82 (0.06)	< .01	0.44	[0.39, 0.50]
Shifting-specific	− 0.02 (0.08)	.77	0.98	[0.83, 1.15]	0.01 (0.08)	.91	1.01	[0.86, 1.18]	− 0.01 (0.08)	.90	0.99	[0.84, 1.17]
Updating-specific	0.10 (0.08)	.19	1.11	[0.95, 1.29]	0.11 (0.07)	.11	1.12	[0.98, 1.28]	0.06 (0.07)	.41	1.06	[0.93, 1.21]
Common EF	0.01 (0.07)	.89	1.01	[0.88, 1.16]	0.06 (0.08)	.43	1.06	[0.91, 1.24]	− 0.10 (0.07)	.19	0.91	[0.79, 1.05]
Positive-constructive	0.19 (0.07)	< .01	1.21	[1.06, 1.38]	0.17 (0.07)	.01	1.18	[1.04, 1.34]	0.16 (0.07)	.02	1.17	[1.02, 1.34]
Guilty-dysphoric	− 0.02 (0.06)	.79	0.98	[0.87, 1.11]	0.06 (0.06)	.34	1.06	[0.94, 1.20]	− 0.03 (0.07)	.69	0.97	[0.86, 1.11]
*Confirmatory interaction model (M*3*)*												
Intercept	− 1.46 (0.06)	< .01	0.23	[0.20, 0.26]	− 0.85 (0.06)	< .01	0.43	[0.38, 0.48]	− 0.81 (0.06)	< .01	0.44	[0.39, 0.50]
Shifting-specific	− 0.02 (0.08)	.80	0.98	[0.84, 1.15]	0.00 (0.08)	.95	1.00	[0.86, 1.17]	− 0.02 (0.08)	.84	0.98	[0.83, 1.16]
Updating-specific	0.11 (0.08)	.16	1.12	[0.96, 1.31]	0.12 (0.07)	.09	1.13	[0.98, 1.30]	0.06 (0.07)	.35	1.07	[0.93, 1.22]
Common EF	0.01 (0.07)	.87	1.01	[0.88, 1.17]	0.07 (0.08)	.41	1.07	[0.91, 1.25]	− 0.09 (0.07)	.21	0.91	[0.79, 1.06]
Positive-constructive	0.19 (0.07)	< .01	1.21	[1.06, 1.39]	0.17 (0.06)	.01	1.18	[1.04, 1.34]	0.16 (0.07)	.02	1.17	[1.02, 1.33]
Guilty-dysphoric	− 0.01 (0.06)	.85	0.99	[0.88, 1.12]	0.07 (0.06)	.27	1.07	[0.95, 1.21]	− 0.02 (0.06)	.81	0.98	[0.87, 1.12]
Positive-constructive × Common EF	− 0.08 (0.07)	.27	0.93	[0.81, 1.06]	− 0.05 (0.07)	.50	0.95	[0.82, 1.10]	− 0.03 (0.08)	.72	0.97	[0.82, 1.15]
Guilty-dysphoric × Updating-specific	0.07 (0.07)	.32	1.07	[0.94, 1.22]	0.11 (0.07)	.11	1.12	[0.97, 1.28]	0.10 (0.07)	.15	1.10	[0.97, 1.26]
*Exploratory interaction model (M*4*)*												
Intercept	− **1.49 (0.07)**	** < .01**	**0.23**	**[0.20, 0.26]**	− **0.86 (0.07)**	** < .01**	**0.42**	**[0.37, 0.48]**	− 0.82 (0.07)	< .01	0.44	[0.39, 0.51]
Shifting-specific	**0.06 (0.10)**	**.54**	**1.06**	**[0.87, 1.30]**	**0.06 (0.09)**	**.53**	**1.06**	**[0.88, 1.28]**	0.01 (0.09)	.94	1.01	[0.85, 1.20]
Updating-specific	**0.10 (0.07)**	**.17**	**1.11**	**[0.96, 1.28]**	**0.11 (0.07)**	**.12**	**1.12**	**[0.97, 1.28]**	0.06 (0.07)	.37	1.06	[0.93, 1.22]
Common executive functioning	− **0.01 (0.07)**	**.94**	**0.99**	**[0.86, 1.15]**	**0.05 (0.08)**	**.50**	**1.05**	**[0.90, 1.23]**	− 0.10 (0.07)	.16	0.90	[0.78, 1.04]
Positive-constructive	**0.17 (0.07)**	** < .01**	**1.19**	**[1.04, 1.35]**	**0.15 (0.07)**	**.02**	**1.16**	**[1.02, 1.32]**	0.14 (0.07)	.04	1.16	[1.01, 1.32]
Guilty-dysphoric	− **0.03 (0.06)**	**.60**	**0.97**	**[0.86, 1.09]**	**0.06 (0.06)**	**.38**	**1.06**	**[0.93, 1.20]**	− 0.02 (0.07)	.72	0.98	[0.86, 1.11]
Positive-constructive × Shifting-specific	**0.08 (0.08)**	**.35**	**1.08**	**[0.92, 1.27]**	**0.07 (0.08)**	**.40**	**1.07**	**[0.91, 1.27]**	0.07 (0.09)	.41	1.08	[0.90, 1.29]
Positive-constructive × Updating-specific	**0.03 (0.09)**	**.74**	**1.03**	**[0.86, 1.24]**	**0.05 (0.08)**	**.57**	**1.05**	**[0.89, 1.23]**	− 0.02 (0.10)	.83	0.98	[0.81, 1.19]
Positive-constructive × Common EF	− **0.09 (0.07)**	**.24**	**0.92**	**[0.80, 1.06]**	− **0.07 (0.09)**	**.44**	**0.93**	**[0.79, 1.11]**	− 0.03 (0.10)	.75	0.97	[0.80, 1.18]
Guilty-dysphoric × Shifting-specific	**0.31 (0.09)**	** < .01**	**1.37**	**[1.15, 1.63]**	**0.21 (0.09)**	**.02**	**1.24**	**[1.03, 1.48]**	0.09 (0.09)	.31	1.10	[0.92, 1.32]
Guilty-dysphoric × Updating-specific	**0.16 (0.07)**	**.02**	**1.17**	**[1.02, 1.34]**	**0.16 (0.07)**	**.02**	**1.18**	**[1.02, 1.36]**	0.15 (0.07)	.04	1.16	[1.00, 1.34]
Guilty-dysphoric × Common EF	− **0.16 (0.07)**	**.02**	**0.85**	**[0.74, 0.97]**	− **0.05 (0.08)**	**.57**	**0.95**	**[0.81, 1.12]**	− 0.06 (0.08)	.45	0.94	[0.79, 1.11]

Model comparisons	Δ*χ*^2^	*P*			Δ*χ*^2^	*p*			Δ*χ*^2^	*p*		
*M*2 vs. *M*1 (df = 5)	11.12	.05			12.69	.03			8.44	.13		
*M*3 vs. *M*2 (df = 2)	2.32	.31			3.43	.18			2.36	.31		
*M*4 vs. *M*2 (df = 6)	**23.17**	** < .01**			**13.30**	**.04**			6.12	.41		
*M*4 vs. *M*1 (df = 11)	**34.29**	** < .01**			**25.99**	** < .01**			14.56	.20		

Endorsed models in bold. *N*_individuals_ = 187. *N*moments = 10,295. TUT, task-unrelated thought; SITUT, Stimulus-independent and task-unrelated thought; SIT, Stimulus-independent thought

In contrast to the negative two-way interaction between common executive functioning ability and the guilty-dysphoric style on SITUTs, the other two two-way interactions were in the positive direction. First, an increase in the guilty-dysphoric style was associated with a more positive slope of shifting-specific ability on SITUTs (see Fig. 4). Simple slopes analysis indicated that among those with a greater guilty-dysphoric style (+ 1 SD), shifting-specific ability positively predicted SITUTs, *B* = 0.38 (SE = 0.15), *p* = 0.01, whereas among those with a lower style (− 1 SD) shifting-specific ability negatively predicted SITUTs, *B* = − 0.25 (SE = 0.10), *p* = 0.02. Second, the interaction between updating-specific ability and guilty-dysphoric was also in the positive direction, contrary to our predictions. Simple slopes analysis (Fig. 4) indicated that updating-specific ability positively predicted SITUTs among those with a greater guilty-dysphoric style, *B* = 0.26 (SE = 0.10), *p* < 0.01, but not significantly for those with a lower guilty-dysphoric style, *B* = − 0.06 (SE = 0.10), *p* = 0.58.

With mind wandering defined as TUTs, the results were similar to those observed with SITUTs but with one notable inconsistency (see Table 5). Common executive functioning ability and guilty-dysphoric style did not significantly interact on TUTs. As with SITUTs, shifting-specific and updating-specific abilities significantly interacted with guilty-dysphoric style on TUTs, and the full model with all interaction terms significantly outperformed the others. By contrast, with mind wandering defined as SITs, no model outperformed the null model.

As in the concentration analysis we evaluated a post hoc multinomial model to distinguish SITUTs, external distractions, task-related interferences, and on-task focus with the latter as the reference category. The interaction between guilty-dysphoric style and shifting-specific ability was only significantly observed for SITUTs, not for external distractions, or task-related interferences (Additional file 1: Table S3). Furthermore, with SITUTs as the baseline, there was a two-way interaction between guilty-dysphoric style and shifting on external distractions, *B* = − 0.25 (0.12), *p* = 0.03, and task-related interferences, *B* = − 0.37 (0.11), *p* < 0.01, suggesting that shifting and guilty-dysphoric style relate differently to SITUTs and each of the other categories. This suggests that as the guilty-dysphoric style increases, shifting predicts greater tendency to engage in internally oriented task-irrelevant thoughts rather than externally oriented distractions or task-relevant thoughts. The results were less clear for updating and common executive functioning (Additional file 1: Table S3). Table 6 summarizes the results concerning our predictions.

**Table 6:** Summary of results concerning predictions of mind wandering (MW) as a function of concentration and affective daydreaming style

*Follow-up on predictions*			
Prediction	Result	Comment	Revised Prediction for further research
Executive functioning (specifically updating) predicts lower MW as concentration increases	Not supported with MW defined as SITUTs (our a priori), but supported with MW defined as TUTs (Kane et al., 2007, 2017)	Post hoc analysis indicated that this association is not SITUT-specific (i.e., EDs, TRIs show similar relations as SITUTs compared to on-task focus)	Updating predicts lower EDs, SITUTs, and TRIs as concentration increases
Executive functioning (specifically updating) predicts lower MW as guilty-dysphoric style increases	Not supported with updating, but supported with common executive functioning	The symmetry span result in Marcusson-Clavertz et al. (2016) may reflect variance due to common executive functioning rather than updating	Common executive functioning predicts lower SITUTs as guilty-dysphoric style increases
Executive functioning (specifically inhibiting) predicts lower MW as positive-constructive style decreases	Not supported, regardless of operationalization of MW or executive functioning	The Stroop result in Marcusson-Clavertz et al. (2016) might have been a false discovery or reflect variance not captured by the cognitive battery in the present study	–

*New prediction*			
A priori exploratory analysis	Result	Comment	Prediction for further research
MW as a function of shifting and daydreaming style	Shifting-specific ability predicted *more* SITUTs as guilty-dysphoric style increased	The opposite effects of shifting-specific and common executive functioning on the slope of guilty-dysphoric style on MW may reflect a stability-flexibility trade-off that is arguably consistent with the neural network model of Herd et al. (2014)	Shifting-specific ability predicts higher SITUTs as guilty-dysphoric style increases

SITUT, stimulus-independent and task-unrelated thought; TUT, task-unrelated thought; ED, external distraction; TRI, task-related interference

## Discussion

This study examined the relations between daily life mind wandering and individual differences in executive functioning and affective styles of mind wandering as well as momentary fluctuations in concentration. Our broad aim was to extend previous research on the interactions between these variables (Kane et al., 2007, 2017; Marcusson-Clavertz et al., 2016) by modelling latent factors of executive functioning and discriminating between different operationalizations of mind wandering. We extracted factor scores in common executive functioning, updating-specific, and shifting-specific abilities based on a bifactor model and used these as predictors of mind wandering operationalized as thoughts that were task-unrelated (i.e., TUTs), stimulus-independent (i.e., SITs), or both (i.e., SITUTs).

**Fig. 5:**
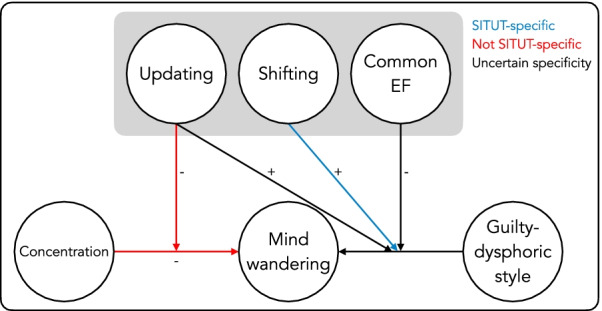
Schematic conceptual depiction of the factors contributing to mind wandering based on the current results. The pluses indicate positive slopes and the minuses indicate negative slopes. For instance, the arrow from concentration to mind wandering indicates that trying harder to concentrate on current activity is associated with a decrease in mind wandering, whereas the arrow from updating pointing at the arrow from concentration to mind wandering indicates that individuals with higher updating show a more strongly negative slope of concentration on mind wandering (i.e., a moderation). The blue arrow indicates that the relation was observed specifically for stimulus-independent and task-unrelated thoughts (SITUTs), whereas the red arrows indicate that the relations were observed for multiple operationalizations of mind wandering, such as external distractions. EF = executive functioning

The first set of results concerns the expected interaction between executive functioning and momentary concentration on daily life mind wandering. Regardless of how mind wandering was operationalized, the associations with executive functioning abilities were small and non-significant, which is consistent with previous research on daily life mind wandering (Kane et al., 2007, 2017; Marcusson-Clavertz et al., 2016). We expected, however, that executive functioning (specifically updating-specific ability) would predict a steeper negative slope on mind wandering as concentration increases. This prediction was not supported with our a priori definition of mind wandering as SITUTs. By contrast, we did observe this cross-level interaction between updating-specific ability and concentration on mind wandering with the latter operationalized as TUTs, which conceptually replicates previous research (Kane et al., 2007, 2017). A post hoc analysis extended these findings by showing that as individuals with greater updating-specific ability try to concentrate harder on a task, they report fewer SITUTs, external distractions, *and* task-related interferences, compared to on-task focus. This suggests that the cross-level interaction between updating-specific ability and concentration on mind wandering is not SITUT-specific (see the red arrows in Fig. 5). In other words, individuals with greater updating couple their perception to external task stimuli to a greater extent as concentration increases, but at the expense of all other mentations.

Our second set of results concerns the expected interaction between affective daydreaming styles and executive functioning abilities on daily life mind wandering. Although we did not conceptually replicate a two-way interaction between affective daydreaming styles and executive functions (Marcusson-Clavertz et al., 2016), exploratory analyses indicated that the guilty-dysphoric style interacted with each of the latent executive functioning factors. One finding that was robust across models indicated that among individuals with a greater guilty-dysphoric daydreaming style, greater shifting-specific ability predicted *more* SITUTs. This finding was specific for SITUTs and significantly different from all other categories, namely external distractions, task-related interferences, and on-task focus (see the blue arrow in Fig. 5). In contrast, common executive functioning predicted a more *negative* slope between the guilty-dysphoric style and SITUTs, which is arguably consistent with previous research (Marcusson-Clavertz et al., 2016). The previous study used the symmetry span task to measure working memory capacity, which is a task that have loaded slightly higher on a common executive functioning factor than a working-memory specific factor (Kane et al., 2016). This may explain why the common executive functioning factor in the present study predicted the slope of this style on mind wandering. Taken together, our results highlight the importance of considering the interplay of specific executive functioning abilities with concentration and affective daydreaming styles to understand the contributing factors of cognition to mind wandering in daily life. It also underlines the importance of distinguishing between task-relatedness and stimulus-dependence in the study of spontaneous cognition.

### Executive functioning and momentary concentration

One of two main results of this study was that updating-specific ability predicts the slope of concentration on TUTs. This interaction effect is consistent with other studies (Kane et al., 2007, 2017). Specifically, updating-specific ability predicted *greater* TUTs during low concentration (see Fig. 3A). Although we cannot draw causal inferences from these data, our interpretation is that as individuals with greater updating skills exert lower effort to concentrate on a task, they have more memory resources available to engage in mentation unrelated to current task stimuli and thus allocate more resources to SITUTs and external distractions (cf., Taatgen et al., 2021). This interpretation is consistent with the global availability hypothesis (Smallwood, 2010), which states that mind wandering is a resource-demanding conscious experience that competes with task-related mentation for the limited space in working memory. Efficient updating skills may thus enable people to engage in more everyday mind wandering at times when they do not need to concentrate on a task (cf., Rummel & Boywitt, 2014). In contrast, on the basis of the cognitive failure hypothesis (McVay & Kane, 2010) one might expect that during moments of high concentration, those with greater updating-specific ability should report significantly less mind wandering, but we did not detect such an association. Insofar as people exert higher effort to concentrate on more demanding tasks, those tasks may be sufficiently demanding to generally prevent high mind wandering regardless of updating capacity, even though the latter predicts actual performance (Marcusson-Clavertz et al., 2020; Rummel & Boywitt, 2014). In addition, our study may have been low powered to detect differences during high concentration because the combination of high concentration and mind wandering is rare. A challenge for ecological momentary assessment designs is the issue of not being able to control task demands or objectively measuring task performance in daily life. Moreover, participants with different executive functioning skills may have different standards for what constitutes a demanding task or high effort or different tendencies to engage in demanding tasks. One promising approach for addressing this challenge is to measure mind wandering both in the laboratory under controlled settings and in daily life and relate the two (Kane et al., 2017).

### Executive functioning and affective daydreaming styles

The second main result of this study concerns the interaction between the guilty-dysphoric style and latent executive functions on mind wandering. Our results suggest that all three factors of executive functioning examined in the present study predict the slope of guilty-dysphoric daydreaming style on mind wandering (i.e., SITUTs). Common executive functioning predicted less mind wandering as guilty-dysphoric style increased, whereas updating-specific and shifting-specific abilities predicted this slope in the *opposite* direction. We conjecture that this discrepancy can be explained through a network model proposing a stability-flexibility trade-off for common executive functioning and shifting-specific ability (Herd et al., 2014), as well as the global availability hypothesis (Smallwood, 2010) and the control-failure hypothesis (McVay & Kane, 2010) of mind wandering.

The control-failure hypothesis postulates that executive cognitive resources prevent mind wandering (McVay & Kane, 2010). There is clear evidence in support of this hypothesis concerning mind wandering during laboratory tasks requiring constant attention (e.g., Kane et al., 2016; Unsworth & McMillan, 2017; Unsworth et al., 2021). However, insofar as an adaptive cognitive system regulates daily life mind wandering differently depending on the context and the content of the experience (Banks et al., 2016; Smallwood & Andrews-Hanna, 2013) people may sometimes allow daily life mind wandering to happen because it can also serve positive functions (Mooneyham & Schooler, 2013). Consistent with this interpretation, the correlation between executive functioning and average mind wandering in daily life appears to be close to zero (Kane et al., 2017; Marcusson-Clavertz et al., 2016). Due to attentional biases to salient, negative affective stimuli (Hankin et al., 2010), guilty-dysphoric mind wandering may require greater inhibitory control. Insofar as the common executive functioning factor extracted in the present study reflects goal maintenance abilities (i.e., the capacity to bias attention towards goal-related stimuli), we speculate that greater biasing towards current activity is needed more often in the face of guilty-dysphoric mind wandering. This could be the reason why common executive functioning predicted lower mind wandering as this style increased.

In contrast to common executive functioning, shifting-specific performance may measure efficiency in goal replacement (Friedman & Miyake, 2017). This ability may be due to efficient clearing of goal representations in working memory by basal ganglia modulation of prefrontal cortex activity or weak goal representations in prefrontal cortex in the first place (Herd et al., 2014). The ability could facilitate the initiation (as well as the termination) of a mind wandering episode insofar as the latter represents a shift from one goal (current activity) to another (an unrelated personal concern). The exploratory finding that shifting-specific ability predicts *greater* mind wandering among people with a high guilty-dysphoric style is consistent with the argument that people with greater goal replacement skills can more frequently switch from on-task mentation to salient off-task mentation, such as guilty-dysphoric mind wandering. That is, a tendency to easily switch goals might lead to increased activations of salient, personal concerns (e.g., guilty-dysphoric mind wandering) during everyday activities. If a person is predisposed to engage in highly salient guilty-dysphoric mind wandering, shifting-specific abilities may facilitate decoupling perception from the here and now to SITUTs. Research on the related concept of maladaptive daydreaming indicates that it has quasi-addictive qualities and that people have strong yearnings for such mentation (Soffer-Dudek et al., 2021).

Updating-specific ability, on the other hand, may measure the efficiency of retrieving information from long term memory and the capacity to actively maintain and manipulate this information in consciousness (Friedman & Miyake, 2017; Verschooren et al., 2021). Our two updating-specific findings suggest that this ability predicts more mind wandering when (a) effort to concentrate on the current activity is low and (b) the guilty-dysphoric style is high. When concentration is low, representations related to the current activity should not be strongly biased and the person should be less constrained to their current activity. Insofar as guilty-dysphoric mind wandering is highly salient it may be easily activated in such circumstances when thought is not severely constrained. Individuals with greater efficiency of episodic memory retrieval and capacity for maintaining a greater amount of information in working memory would have surplus resources available for mind wandering. This interpretation aligns with the global availability hypothesis (Smallwood, 2010), which postulates that mind wandering requires information-processing resources and thus competes with on-task mentation for the limited space in working memory, implying that those with greater resources available could mind wander more frequently. To summarize, among those with greater guilty-dysphoric propensity, common executive functioning processes may be increasingly employed to prevent mind wandering, whereas shifting may facilitate more switches to salient, dysphoric mind wandering, and updating may contribute to the maintenance of such episodes (particularly when concentration is low, see Fig. 5).

Two sets of results are more difficult to explain from this perspective. First, if common executive functioning measures goal maintenance, it is difficult to understand why it did not significantly predict less mind wandering as concentration increases. Rather, we found a large decrease in mind wandering as effort to concentrate increases regardless of common executive functioning ability, suggesting that our sample as a whole was effective at reducing their mind wandering when they concentrated more. In contrast, Kane et al. (2017) observed this interaction between concentration and a factor they termed attention-restraint (including the SART, a number Stroop task, and the antisaccade task) although they evaluated this factor and working memory in separate models. A limitation of our study is that the inhibiting task had moderate loadings on the inhibiting and common executive functioning factors. We may also have had low power to detect such interaction between common executive functioning and concentration as the combination of high concentration and mind wandering is rare. A more general limitation of these experience sampling studies is that they measured concentration through self-report and people with greater executive functioning may require less conscious effort in performing challenging tasks making it difficult to compare individuals varying in this dimension (cf., Naccache et al., 2005). If common executive functioning involves monitoring of goal adherence, it may also be that people scoring low on common executive functioning are less capable of monitoring their stream of consciousness rendering it difficult to indicate whether they were mind wandering or not. Manipulating effort (e.g., providing monetary rewards for strong performance) may be an alternative means to examine the interactions between common executive functions and effort on mind wandering. Perhaps some small amount of mind wandering even during challenging tasks is inevitable or even desirable, and perhaps it is more about how that mind wandering impacts performance. For instance, mind wandering without awareness is negatively related to reading comprehension (Smallwood et al., 2007) and self-reported performance on daily life activities (McVay et al., 2009).

The second set of results that are difficult to interpret are the null results concerning the interaction between the positive-constructive daydreaming style and executive functioning on mind wandering. The borderline significant interaction between this style and inhibition observed in the previous study may thus have been spurious or specific to that particular cognitive task (Marcusson-Clavertz et al., 2016). A limitation of the current study is that we did not discriminate between visuospatial and verbal processes in our modelling of executive functioning. These two storage systems are related but distinct (Baddeley, 1992; Fournier-Vicente et al., 2008). As positive-constructive mind wandering often entails visual imagery whereas guilty-dysphoric typically seems not to (Cardeña & Marcusson-Clavertz, 2016; Marcusson-Clavertz et al., 2016), they may relate differently to individual differences in visuospatial and phonological memory storage. This possibility could be assessed by separating between visuospatial and verbal tasks and relate these to the daydreaming styles.

### Strengths and limitations

A strength of this study is the high ecological validity of the experience sampling methodology which was used to sample mind wandering across multiple activities and contexts. The inclusion of multiple cognitive tasks per construct and the use of factor analysis to extract latent factors also reduce the risk that idiosyncrasies of a particular task confound the results. Despite using different cognitive tasks and slightly different phrasing in the mind wandering prompts than Kane et al. (2017), there are several conceptual replications across our studies, such as working memory updating negatively predicting the slope of concentration on TUTs. However, our study examined a young sample ranging from 18 to 42 years old, which prevents generalizing the results to younger and older age groups, who may exhibit different patterns of executive functioning (Brydges et al., 2014; Hedden & Yoon, 2006) and mind wandering (Moran et al., 2021). Furthermore, although a strength of this study was the multidimensional focus on mind wandering, which has been called for (Christoff et al., 2016; Seli et al., 2018; Smith et al., 2018), it comprised a large number of analyses, inflating the familywise error rate. We attempted to control error rate by testing the significance of the overall multilevel models before interpreting the coefficients of specific predictors and clearly distinguishing between confirmatory, planned exploratory, and post hoc exploratory tests. We did not adjust α for the exploratory analyses and thus those results should be interpreted with more caution.

A limitation is that we measured concentration with a single self-report question. Although responses to this question predicted objective performance in a working memory task, according to our reanalysis of data from a previous study (Marcusson-Clavertz et al., 2020), this question may sometimes be difficult to answer. Unlike cognitive laboratory tasks, which usually require constant attention, many daily life activities can be performed with fluctuating attention (e.g., reading the newspaper, doing the laundry, cooking dinner). In these scenarios effort to concentrate may also fluctuate quickly from one moment to the other and be difficult to summarize quantitatively. In addition, the extent a person might try to concentrate on the current task likely depends on several factors—the difficulty of the task, the cost of making an error, the salience of task stimuli, how absorbed the person already is on the task, etc.—and these reasons might affect the relation between mind wandering and executive functioning. If a person is highly absorbed in a task, concentration could be effortless and this could also complicate answering this question. However, according to a previous study, participants typically report that they are trying to concentrate to a greater extent when they feel more absorbed and perform more attention-demanding and interesting activities (Cardeña & Marcusson-Clavertz, 2016). That this measure has interacted with working memory performance on TUTs in three studies is also promising, but a more nuanced psychometric evaluation could clarify these results and point to potential confounding variables. An alternative laboratory method to corroborate these findings could be to manipulate the extent people try to concentrate on a long, monotonous task by adding rewards for good performance, and examine if updating predicts reductions in TUTs as rewards increase (cf., Rummel & Boywitt, 2014).

Although the executive functioning models showed good fit to our data, it is worth noting that the inhibiting factor showed smaller loadings, and we endorsed the bifactor model instead of the correlated factors model. The inhibiting tasks were chosen to broadly cover attentional restraint (i.e., preventing a prepotent response) and constraint (i.e., resolving interference from visual distractors). This broad selection might have reduced the covariance, particularly for the flanker task, which, in contrast to our other tasks, may tap attentional constraint (c.f., Friedman & Miyake, 2004; Kane et al., 2016). The antisaccade task would be a suitable alternative to the flanker task if the aim is to extract a purer attentional restraint factor (Kane et al., 2016). Another limitation concerning the cognitive battery is that the 2-back task loaded more on the common executive functioning factor than the expected updating factor. In hindsight, a 3-back task would have been a better choice insofar as it places more demands on working memory updating (cf., Kane et al., 2007; Schmiedek et al., 2014). We chose a 2-back version because we were concerned that the task battery could be perceived as too difficult and long for some participants, and a faster 2-back version would also comprise more trials within a limited time window. A higher number of 1-back and 3-back lures would likely place greater demands on updating skills as well. Nevertheless, our results are similar to those of Himi et al. (2019) who used a mix of 2-back and 3-back tasks, which also loaded strongly on the common executive functioning factor.

## Conclusions

To summarize, with increased effort to concentrate on the current task, working memory updating skills predict greater constraint of perception to task stimulus. Consequently, when effort to concentrate on the task is low, greater working memory updating is associated with more time spent on thoughts unrelated to the task and/or independent of current stimulus. The findings from the exploratory analyses suggest that the combination of greater shifting-specific ability and guilty-dysphoric daydreaming style is associated with greater amount of time spent on internally oriented task-irrelevant thoughts. In contrast, greater common executive functioning predicted less mind wandering as the guilty-dysphoric style increases. The opposite slopes of common executive functioning and shifting-specific abilities may reflect a trade-off between the abilities to maintain goals active in the mind and efficiently replacing no-longer-active goals. This research program is admittedly at an early stage, and many predictions received mixed support depending on the operationalizations, but we maintain that the results of this study on how latent executive functioning, concentration, and affective daydreaming style relate to mind wandering can help integrate recent research (Kane et al., 2007, 2017; Marcusson-Clavertz et al., 2016; McVay & Kane, 2009) with theoretical accounts of common executive functioning, working memory, and task-switching (Herd et al., 2014; McVay & Kane, 2010; Smallwood, 2010) and provide promising leads for future research.

## Supplementary Information

**Additional file 1**. Descriptive summaries of cognitive tasks and experience sampling questionnaire and additional correlational and multilevel analyses on experience sampling questionnaire data.

## Data Availability

Data and analytic scripts used for the multilevel and factor analyses in the present paper are available in the Open Science Framework repository (https://osf.io/rzs5d/). Further data can be obtained by reasonable request to the corresponding author.

## Abbreviations

TUT: Task-unrelated thought
SIT: Stimulus-independent thought
SITUT: Stimulus-independent and task-unrelated thought
SIPI: Short imaginal processes inventory
LISAS: Linear integrated speed-accuracy score
RT: Response time
CFI: Comparative fit index
SRMR: Standardized root mean square residual
RMSEA: Root mean square error of approximation

## Footnotes

1 We use mind wandering and daydreaming interchangeably in this paper, but retain the terms usually used to refer to the specific instrument in question.

2 Although the plan was publicly available at the OSF website prior to data collection, due to a mistake it was not formally pre-registered.

3 We retain the terms used in previous research, although “distractions” and “interferences” may be more easily applied to the context of performing laboratory, cognitive tasks that demand continuous attention to external stimuli than daily life activities that allow for fluctuations in attention.

4 For each of the four tasks, difference scores in LISAS and raw mean RT correlated strongly (*r*s > .92), possibly due to the highly accurate responding (at least 89% correct responses across all conditions and tasks).
