# Supplementary material for: The contribution of latent factors of executive functioning to mind wandering: an experience sampling study
Source: Cogn Res Princ Implic. 2022 Apr 25;7:34. doi: 10.1186/s41235-022-00383-9 (PMC9038971; doi:10.1186/s41235-022-00383-9)
Supplement: Supplementary file 1 — Additional file 1. Descriptive summaries of cognitive tasks and experience sampling questionnaire and additional correlational and multilevel analyses on experience sampling questionnaire data. [file 41235_2022_383_MOESM1_ESM.docx]

**Supplementary material for the manuscript**

**“The contribution of latent factors of executive functioning to mind wandering: An experience sampling study”^[[1]](#footnote-1)^**

Table S1 and S2 show descriptive summaries of cognitive tasks and experience sampling reports. Table S3 shows the results of the *post hoc* multinomial analyses examining whether cognitive factors and affective daydreaming styles predict stimulus-independent and task-unrelated thoughts (SITUTs), external distractions, task-related interferences, and on-task focus (the latter is used as a reference category).

**Table S1** Descriptive summary of cognitive tasks (full sample)

| **Task** | **Variable** | ***N*** | ***M*** | ***SD*** | **Minimum** | **Maximum** | **Skewness** | **Kurtosis** |
| --- | --- | --- | --- | --- | --- | --- | --- | --- |
| Go/ No-go | Proportion of correct No-go trials | 194 | .69 | .13 | .26 | .92 | -0.55 | -0.20 |
| Go/ No-go | Sum of anticipations | 194 | 0.90 | 1.75 | 0.00 | 10.00 | 2.58 | 7.56 |
| Go/ No-go | Sum of omissions | 194 | 1.14 | 2.00 | 0.00 | 11.00 | 2.53 | 6.97 |
| Go/ No-go | RT coefficient of variability (ms) | 194 | 0.21 | 0.05 | 0.12 | 0.43 | 1.06 | 1.51 |
| Flanker | RT on congruent trials (ms) | 196 | 356 | 43 | 282 | 545 | 1.19 | 2.41 |
| Flanker | RT on incongruent trials (ms) | 196 | 412 | 60 | 286 | 822 | 2.14 | 11.20 |
| Flanker | Proportion of correct congruent trials | 196 | .98 | .03 | .70 | 1.00 | -5.56 | 43.60 |
| Flanker | Proportion of correct incongruent trials | 196 | .91 | .08 | .60 | 1.00 | -1.15 | 0.82 |
| Flanker | LISAS switchcost | 196 | 70 | 38 | -5 | 351 | 2.79 | 16.20 |
| Stop-signal | Stop-signal RT (ms) | 190 | 250 | 30 | 159 | 356 | 0.04 | 0.53 |
| Stop-signal | Respond-signal RT (ms) | 190 | 464 | 101 | 307 | 939 | 1.71 | 4.10 |
| 2-back | Proportion of correct non-match trials | 195 | .84 | .16 | .07 | .99 | -2.83 | 9.04 |
| 2-back | Proportion of correct match trials | 195 | .62 | .18 | .01 | .97 | -0.33 | -0.34 |
| Letter memory | Sum of correctly recalled letters | 193 | 30.80 | 6.56 | 12 | 44 | -0.42 | 0.05 |
| Keep track | Sum of correctly recalled words | 190 | 25.60 | 3.93 | 13 | 35 | -0.51 | 0.20 |
| Number-letter | RT on repeat trials (ms) | 196 | 853 | 213 | 521 | 1824 | 1.54 | 3.75 |
| Number-letter | RT on switch trials (ms) | 196 | 1260 | 441 | 625 | 3910 | 2.01 | 7.15 |
| Number-letter | Proportion of correct repeat trials | 196 | .94 | .07 | .52 | .98 | -4.05 | 19.00 |
| Number-letter | Proportion of correct switch trials | 196 | .93 | .08 | .44 | 1.00 | -3.76 | 19.30 |
| Number-letter | LISAS switchcost | 196 | 451 | 415 | -317 | 2957 | 2.59 | 9.88 |
| Local-global | RT on repeat trials (ms) | 195 | 872 | 202 | 449 | 1435 | 0.58 | -0.14 |
| Local-global | RT on switch trials (ms) | 195 | 1232 | 274 | 580 | 2020 | 0.34 | -0.23 |
| Local-global | Proportion of correct repeat trials | 195 | .94 | .05 | .76 | 1.00 | -1.86 | 3.98 |
| Local-global | Proportion of correct switch trials | 195 | .90 | .08 | .63 | 1.00 | -1.37 | 2.14 |
| Local-global | LISAS switchcost | 195 | 404 | 186 | -60 | 1391 | 1.14 | 4.11 |
| Color-Shape | RT on repeat trials (ms) | 198 | 536 | 108 | 294 | 1001 | 0.75 | 0.96 |
| Color-Shape | RT on switch trials (ms) | 198 | 641 | 142 | 345 | 1173 | 0.64 | 0.42 |
| Color-Shape | Proportion of correct repeat trials | 198 | .94 | .05 | .68 | 1.00 | -2.54 | 9.41 |
| Color-Shape | Proportion of correct switch trials | 198 | .89 | .08 | .63 | 1.00 | -1.04 | 1.05 |
| Color-Shape | LISAS switchcost | 198 | 133 | 88 | -32 | 551 | 1.12 | 2.71 |

**Table S2** Descriptive summary of experience-sampling items (person means; full sample, *N* = 193)

| **#** | **Question** | **Response coding** | ***M*** | ***SD*** | **Min** | **Max** | **Skewness** | **Kurtosis** |
| --- | --- | --- | --- | --- | --- | --- | --- | --- |
| 1 | Were you thinking about the activity you were doing? | 1 = *no, something else* [TUT], 0=*yes, activity* | 0.33 | 0.17 | 0 | 1 | 0.63 | 0.83 |
| 2 | Were you thinking about something in the immediate surroundings? | 1 = *no, something else* [SIT],  0 = *yes, surroundings* | 0.33 | 0.17 | 0 | 0.85 | 0.55 | 0.15 |
| 3 | Were you in control of/guiding your thoughts? | 0 (*not at all*) to 1 (*fully*) | 0.56 | 0.16 | 0.11 | 0.93 | -0.13 | 0.07 |
| 4 | How aware were you of what you were thinking about? | 0 (*not at all*) to 1 (*fully*) | 0.62 | 0.14 | 0.14 | 0.97 | -0.22 | 0.16 |
| 5 | How well can you remember what you were thinking about? | 0 (*not at all*) to 1 (*very*) | 0.67 | 0.13 | 0.28 | 0.96 | -0.2 | -0.12 |
| 6 | How much were you trying to concentrate on the activity? | 0 (*not at all*) to 1 (*a lot*) | 0.48 | 0.13 | 0.09 | 0.91 | 0.14 | 0.58 |
| 7 | Were you distracted by things in the immediate surroundings? | 0 (*not at all*) to 1 (*a lot*) | 0.42 | 0.14 | 0.1 | 0.78 | 0.05 | -0.13 |
| 8 | Were you having difficulty maintaining concentration on what you were doing? | 0 (*not at all*) to 1 (*a lot*) | 0.4 | 0.12 | 0.05 | 0.74 | -0.13 | -0.06 |
| 9 | Were you feeling…? | 0 (*passive*) to 1 (*active*) | 0.56 | 0.1 | 0.28 | 0.83 | 0.01 | -0.05 |
| 10 | Were you feeling…? | 0 (*sad*) to 1 (*happy*) | 0.64 | 0.1 | 0.32 | 0.96 | 0.22 | 0.54 |
| 11 | Were you feeling…? | 0 (*anxious*) to 1 (*calm*) | 0.67 | 0.13 | 0.29 | 0.95 | 0.01 | -0.36 |
| 12 | Were you feeling…? | 0 (*bored*) to 1 (*amused*) | 0.6 | 0.1 | 0.28 | 0.9 | 0.24 | 0.68 |
| 13 | In the last 10 min, were you having difficulty shifting focus between activities? | 0 (*not at all*) to 1 (*a lot*) | 0.32 | 0.15 | 0 | 0.67 | -0.11 | -0.6 |
| 14 | In the last 10 min, were you having difficulty changing the way you thought about something? | 0 (*not at all*) to 1 (*a lot*) | 0.31 | 0.16 | 0.01 | 0.73 | -0.09 | -0.82 |

*Note*. TUT = task-unrelated thought. SIT = Stimulus-independent thought. All item response scales were 100-point VAS scales except the first two.

**Table S3** Multinomial hierarchical linear modelling with stimulus-independent and task-unrelated thoughts (SITUTs), external distractions, and task-related interferences compared to on-task focus (final sample, *N* = 187)

|  | **SITUTs vs. on-task focus** | | | | **External distractions vs. on-task focus** | | | | **Task-related interferences vs. on-task focus** | | | |
| --- | --- | --- | --- | --- | --- | --- | --- | --- | --- | --- | --- | --- |
|  | ***B(SE)*** | ***p*** | ***OR*** | **95% *CI*** | ***B(SE)*** | ***P*** | ***OR*** | **95% *CI*** | ***B(SE)*** | ***p*** | ***OR*** | **95% *CI*** |
| Intercept | -1.07 (0.07) | <.01 | 0.34 | [0.30, 0.39] | -1.79 (0.09) | <.01 | 0.17 | [0.14, 0.20] | -1.94 (0.11) | <.01 | 0.14 | [0.12, 0.18] |
| Shifting-specific | 0.04 (0.08) | .60 | 1.05 | [0.89, 1.24] | 0.02 (0.12) | .87 | 1.02 | [0.81, 1.28] | -0.10 (0.14) | .49 | 0.91 | [0.69, 1.20] |
| Updating-specific | 0.12 (0.08) | .11 | 1.13 | [0.97, 1.31] | 0.07 (0.10) | .47 | 1.07 | [0.88, 1.30] | -0.04 (0.11) | .72 | 0.96 | [0.78, 1.19] |
| Common EF | -0.01 (0.07) | .90 | 0.99 | [0.86, 1.15] | 0.15 (0.09) | .10 | 1.16 | [0.97, 1.39] | -0.14 (0.11) | .22 | 0.87 | [0.70, 1.09] |
| Positive-constructive | 0.18 (0.07) | .01 | 1.20 | [1.04, 1.39] | 0.06 (0.09) | .50 | 1.06 | [0.89, 1.27] | 0.05 (0.11) | .62 | 1.06 | [0.85, 1.31] |
| Guilty-dysphoric | 0.00 (0.08) | .98 | 1.00 | [0.86, 1.16] | 0.18 (0.09) | .04 | 1.20 | [1.01, 1.43] | 0.05 (0.11) | .63 | 1.05 | [0.85, 1.31] |
| Positive-constructive × Shifting-specific | 0.11 (0.11) | .33 | 1.11 | [0.90, 1.38] | 0.08 (0.12) | .53 | 1.08 | [0.85, 1.38] | 0.01 (0.14) | .94 | 1.01 | [0.77, 1.32] |
| Positive-constructive × Updating-specific | 0.02 (0.07) | .77 | 1.02 | [0.90, 1.16] | 0.03 (0.11) | .80 | 1.03 | [0.83, 1.28] | -0.11 (0.08) | .19 | 0.90 | [0.77, 1.05] |
| Positive-constructive × Common EF | -0.09 (0.07) | .22 | 0.92 | [0.80, 1.06] | -0.04 (0.09) | .68 | 0.96 | [0.81, 1.15] | 0.05 (0.10) | .62 | 1.05 | [0.86, 1.28] |
| Guilty-dysphoric × Shifting-specific | 0.25 (0.09) | <.01 | 1.29 | [1.07, 1.54] | 0.00 (0.12) | 1.00 | 1.00 | [0.79, 1.27] | -0.12 (0.13) | .35 | 0.88 | [0.68, 1.14] |
| Guilty-dysphoric × Updating-specific | 0.18 (0.09) | .04 | 1.20 | [1.01, 1.43] | 0.14 (0.10) | .15 | 1.15 | [0.95, 1.40] | 0.14 (0.12) | .24 | 1.15 | [0.91, 1.45] |
| Guilty-dysphoric × Common EF | -0.12 (0.08) | .16 | 0.89 | [0.75, 1.05] | 0.05 (0.09) | .61 | 1.05 | [0.88, 1.25] | 0.01 (0.10) | .89 | 1.01 | [0.83, 1.24] |

*Note. N* _moments_ = 10,295. EF = executive functioning. SITUTs = stimulus-independent and task-unrelated thoughts.

Table S3 shows that shifting-specific ability predicted more mind wandering (stimulus-independent and task-unrelated thoughts; SITUTs) among those with greater guilty-dysphoric daydreaming style. In contrast, shifting-specific ability did not predict external distractions or task-related interferences as a function of the guilty-dysphoric style.

Below we report the analysis of the six experience-sampling items designed to tap similar constructs to the inhibiting, updating, shifting factors (measured with the cognitive battery), as detailed in the original analysis plan (see Aim 2; <https://osf.io/hk4fc/>). However, because we endorsed the bifactor model of the cognitive battery partly due to a very high factor correlation between inhibiting and updating, we instead report the associations using the factor scores from this bifactor model (i.e., *common executive functioning, shifting-specific*, and *updating-specific*). The descriptive statistics for the six experience sampling items are reported in Table S2 and have number 4 (aware of thought), 5 (remember thought), 7 (distracted by surroundings), 8 (difficulty maintain concentration), 13 (difficulty shift activity), and 14 (difficulty change perspective) in the questionnaire. We planned to create mean scores of items 4 and 5 (*perceived awareness*), 7 and 8 (*perceived distractibility*), 13 and 14 (*perceived difficulty mental set shifting*). The correlations between person means of these six items, their two-item scales, and the three executive functioning factor scores is reported in Table S4. As can be seen in Table S4, greater factor scores in updating-specific and common executive functioning ability correlated with lower perceived difficulty mental set shifting.

We tested the three-level multivariate model detailed in the analysis plan, in which each of the three executive functions (shifting-specific, updating-specific, common executive functioning) were used as predictors and the three experience sampling scales (perceived awareness, perceived distractibility, perceived difficulty mental set shifting) were used as outcomes. Level 1 was experience-sampling scale (perceived distractibility and perceived difficulty mental set shifting were reversed here so that all three scales were scored in the same direction), level 2 was moment (*n* = 10220), and Level-3 was person (*N* = 187).

This multivariate model was significant χ^2^ (3) = 10.06, *p* = .018, indicating that the cognitive battery predicted experience sampling responses. However, no specific factor significantly predicted overall responses to these items (i.e., perceived mental ability). The closest was common executive functioning that had a non-significant positive slope on perceived mental ability, *B* = 0.02, *SE* = 0.01, *t*(183) = 1.85, *p* = .063. The slope was also non-significant positive for updating-specific, *B* = 0.01, *SE* = 0.01, *t*(183) = 1.44, *p* = .150, but non-significant negative for shifting-specific ability as predictor of perceived mental ability, *B* = -0.01, *SE* = 0.01, *t*(183) = -1.43, *p* = .152.

Analyzing the three experience sampling scales separately indicated only significant associations for perceived difficulty mental set shifting. Contrary to our expectations, this outcome was not predicted by the shifting-specific ability, *B* = 0.01, *SE* = 0.01, *t*(183) = 1.39, *p* = .166. Instead, better common executive functioning predicted lower perceived difficulty mental set shifting, *B* = -0.03, *SE* = 0.01, *t*(183) = -2.85, *p* = .005 Updating-specific ability also predicted lower perceived difficulty mental set shifting, *B* = -0.02, *SE* = 0.01, *t*(183) = -2.61, *p* = .010 (see also the correlations in Table S4). To summarize, individuals with greater common executive function and updating-specific performances in the laboratory generally reported feeling lower difficulty alternating between activities and changing the way they thought about things in daily life.

**Table S4.** Correlations between scores on latent cognitive factors and experience sampling responses

| Variable | 1 | 2 | 3 | 4 | 5 | 6 | 7 | 8 | 9 | 10 | 11 | 12 |
| --- | --- | --- | --- | --- | --- | --- | --- | --- | --- | --- | --- | --- |
| 1. Shifting-specific ability |  |  |  |  |  |  |  |  |  |  |  |  |
| 2. Updating-specific ability | -.16***** |  |  |  |  |  |  |  |  |  |  |  |
| 3. Common executive functioning ability | .22****** | .32****** |  |  |  |  |  |  |  |  |  |  |
| 4. Aware of thought | -.05 | -.07 | -.06 |  |  |  |  |  |  |  |  |  |
| 5. Remember thought | -.15***** | .04 | .04 | .56****** |  |  |  |  |  |  |  |  |
| 6. Distracted by surroundings | .07 | -.04 | .06 | -.44****** | -.55****** |  |  |  |  |  |  |  |
| 7. Difficulty maintain concentration | .09 | -.07 | -.07 | -.31****** | -.53****** | .77****** |  |  |  |  |  |  |
| 8. Difficulty shift activity | .11 | -.18***** | -.22****** | -.24****** | -.40****** | .51****** | .65****** |  |  |  |  |  |
| 9. Difficulty change perspective | .04 | -.27****** | -.22****** | -.26****** | -.37****** | .41****** | .61****** | .76****** |  |  |  |  |
| 10. Perceived awareness | -.11 | -.02 | -.02 | .89****** | .87****** | -.56****** | -.47****** | -.35****** | -.36****** |  |  |  |
| 11. Perceived distractibility | .08 | -.05 | .00 | -.40****** | -.57****** | .95****** | .93****** | .62****** | .53****** | -.55****** |  |  |
| 12. Perceived difficulty mental set shifting | .08 | -.24****** | -.24****** | -.27****** | -.41****** | .49****** | .67****** | .93****** | .94****** | -.38****** | .61****** |  |

Note. The first three variables are executive functioning factor scores based on the cognitive battery, whereas the others are experience-sampling variables (person means). *N* = 187 for all the correlation with executive functioning factors and *N* = 192 for all the correlations of pairs of experience-sampling questionnaire responses. Perceived awareness is the mean of variable 4 and 5. Perceived distractibility is the mean of variables 6 and 7. Perceived difficulty mental set shifting is the mean of variables 8 and 9.

1. Corresponding author: [david.marcussonclavertz@lnu.se](mailto:david.marcussonclavertz@lnu.se) (dmc2617@gmail.com), Linnaeus University, Department of psychology, Hus L Trummenvägen 11, 391 82, Växjö, Sweden [↑](#footnote-ref-1)
